# Engineered Hydrogels for Organoid Models of Human Nonalcoholic Fatty Liver Disease

**DOI:** 10.1002/advs.202417332

**Published:** 2025-05-14

**Authors:** Yueming Liu, Aidan E. Gilchrist, Patrik K. Johansson, Yuan Guan, Jaydon D. Deras, Yu‐Chung Liu, Sofia Ceva, Michelle S. Huang, Renato S. Navarro, Annika Enejder, Gary Peltz, Sarah C. Heilshorn

**Affiliations:** ^1^ Department of Materials Science and Engineering Stanford University Stanford CA 94305 USA; ^2^ Department of Biomedical Engineering University of California Davis CA 95616 USA; ^3^ Department of Anesthesiology Pain and Perioperative Medicine Stanford University School of Medicine Stanford CA 94305 USA; ^4^ Department of Chemical Engineering Stanford University Stanford CA 94305 USA; ^5^ Department of Biomedical Engineering University of Michigan Ann Arbor MI 48109 USA; ^6^ Department of Biology Stanford University Stanford CA 94305 USA

**Keywords:** engineered hydrogels, hepatic organoids, lipid accumulation, matrix stiffening, mechanosignaling

## Abstract

Nonalcoholic fatty liver disease (NAFLD) is characterized by increased lipid accumulation and excessive deposition of extracellular matrix (ECM) that results in tissue stiffening. The potential interplay between matrix stiffness and hepatocyte lipid accumulation during NAFLD has not been established. Here, an in vitro NAFLD model is developed using chemically defined, engineered hydrogels and human induced pluripotent stem cell‐derived hepatic organoids (HOs). Specifically, dynamic covalent chemistry crosslinking, along with transient small molecule competitors, are used to create dynamic stiffening hydrogels that enable the reproducible culture of HOs. Within matrices that mimic the stiffness of healthy to diseased tissue (≈1–6 kPa), lipid droplet accumulation in HOs is triggered by exposure to an NAFLD‐associated free fatty acid. These NAFLD model suggests that higher stiffness microenvironments result in increased hepatic lipid droplet accumulation, increased expression of fibrosis markers, and increased metabolic dysregulation. By targeting the ROCK mechanosignaling pathway, the synergy between matrix stiffness and lipid droplet accumulation is disrupted. The in vitro model of NAFLD has the potential to understand the role of mechanosignaling in disease progression and identify new pathways for therapeutic intervention.

## Graphical abstract

Nonalcoholic fatty liver disease (NAFLD) is characterized by lipid accumulation and extracellular matrix stiffening. An in vitro NAFLD model is developed using engineered hydrogels (*G*′ ≈ 1–6 kPa) and iPSC‐derived hepatic organoids. It is demonstrated that higher stiffness can increase lipid droplet accumulation, fibrosis, and metabolic dysregulation, which can be reversible with ROCK inhibitor Y27632. This model highlights mechanosignaling's role in NAFLD progression and potential therapeutic targets.

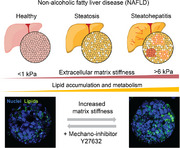

## 1 Introduction

Nonalcoholic fatty liver disease (NAFLD) is a chronic condition that is the leading cause for end stage liver disorders.^[^ ^1^, ^2^ ^]^ Affecting 32% of the adult population in the world, NAFLD has only one available FDA‐approved treatment.^[^ ^3^, ^4^, ^5^, ^6^ ^]^ NAFLD is characterized by the onset of excess lipid accumulation in hepatocytes, termed steatosis, which can progress to Nonalcoholic steatohepatitis (NASH), and potentially to end‐stage cirrhosis and hepatocellular carcinoma (HCC).^[^ ^7^, ^8^, ^9^ ^]^ Another important hallmark of NAFLD is excessive extracellular matrix (ECM) deposition, which causes fibrosis and a continuous stiffening of the liver microenvironment.^[^ ^10^, ^11^ ^]^ Mechanistic studies of NAFLD progression are complicated by the concurrence of multiple NAFLD symptoms: (i) elevated lipid accumulation from circulating free fatty acids (FFA), (ii) fibrotic liver stiffening, and (iii) metabolic dysregulation.^[^ ^12^, ^13^ ^]^ To address this challenge, here we introduce an engineered human model of NAFLD that enables independent control of matrix stiffness and FFA presentation. While ECM mechanical properties are known to affect hepatocyte metabolic function and to activate hepatic stellate cells,^[^ ^14^, ^15^, ^16^, ^17^ ^]^ the potential causative relationship between matrix stiffening and hepatocyte lipid accumulation has not been demonstrated.

Studies of ECM stiffness during NAFLD pathogenesis have provided suggestive evidence that mechanosignaling may impact lipid accumulation;^[^ ^18^ ^]^ however, they have been hindered by inadequate in vivo and in vitro models.^[^ ^19^, ^20^ ^]^ For example, human studies on NAFLD are constrained by limited access to liver tissue, requiring indirect assessments such as serum biomarkers, elastography, or tracers.^[^ ^21^ ^]^ Additionally, these studies are highly complex due to the diverse influences of diet, genetics, and environmental factors.^[^ ^21^ ^]^ Animal models of NAFLD capture histological features of NAFLD, but do not demonstrate clinically relevant disease progression.^[^ ^22^, ^23^, ^24^, ^25^ ^]^ To date, most in vitro studies into mechanosignaling pathways and hepatocyte metabolism have been conducted on 2D substrates,^[^ ^14^, ^15^, ^16^, ^17^, ^20^ ^]^ which are not representative of the 3D liver tissue microenvironment. As an alternative, 3D in vitro models have been used to study diet‐related lipid accumulation and liver fibrosis,^[^ ^19^, ^26^ ^]^ although the role of matrix stiffness on hepatocyte lipid accumulation has not been explored.

Engineered matrices with tunable mechanical properties are promising in vitro platforms for 3D culture to study the interplay between matrix stiffness and cell response.^[^ ^27^, ^28^, ^29^ ^]^ Current 3D in vitro models of NAFLD include single cells encapsulated within a matrix or organoids cultured with a matrix or liquid suspension.^[^ ^30^, ^31^, ^32^, ^33^, ^34^ ^]^ Single cell cultures lack the complex structure and biological function of the in vivo tissue. However, organoid models have typically relied on animal derived matrices, which have significant batch‐to‐batch variability and do not allow for easily changing the stiffness without altering protein concentrations.^[^ ^35^, ^36^ ^]^ As an alternative, engineered matrices with well‐defined biochemical and biophysical cues (e.g., adhesive ligand density and stiffness) provide a more reproducible and tunable biomaterial platform for 3D in vitro models.^[^ ^37^, ^38^, ^39^, ^40^ ^]^ For example, chemically defined matrices with different mechanical properties have been successfully used to support the culture of human liver organoids^[^ ^41^, ^42^, ^43^ ^]^ and to study mechanotransduction in a mouse liver organoid model of fibrosis.^[^ ^42^ ^]^

Here, we establish a human NAFLD model using chemically defined, engineered hydrogels and human induced pluripotent stem cell (hiPSC)‐derived hepatic organoids (HOs) to study how matrix stiffness impacts hepatocyte lipid accumulation, a key indicator of NAFLD disease progression. iPSC‐derived HOs show structural, gene‐transcriptional, and functional similarities to liver tissue and consistently regenerate from single cells, enabling reproducible and rigorous experimental design.^[^ ^44^, ^45^ ^]^ Moreover, they have previously been used to develop models of liver disease, including fibrosis.^[^ ^46^, ^47^ ^]^ We develop engineered matrices with three distinct stiffness regimes that mimic the liver tissue microenvironment in healthy, early‐onset, and advanced stages of NAFLD. Consistent with previous reports, we observe that liver organoid cultures are less likely to form in high stiffness matrices,^[^ ^42^ ^]^ especially those that recapitulate advanced fibrosis. Thus, to overcome this obstacle, we design a dynamic stiffening matrix that is initially soft to support HO growth and then stiffens over time to mimic fibrosis. Our results demonstrate that a high stiffness microenvironment drives higher hepatic lipid droplet accumulation and increased lipid metabolism in the presence of FFA. Treating the organoids with inhibitors of mechano‐signaling moderates lipid droplet accumulation, suggesting that mechano‐therapeutics could be a potential way to slow down NAFLD progression. Altogether, biomimetic, dynamic stiffening hydrogels with human HOs offer a new platform to study the mechano‐signaling mechanisms underlying NAFLD and have future potential as a novel drug screening platform.

## 2 Results and Discussion

### 2.1 Small Molecule Competitor to Achieve Dynamic Stiffening Hydrogels

**Figure 1:**
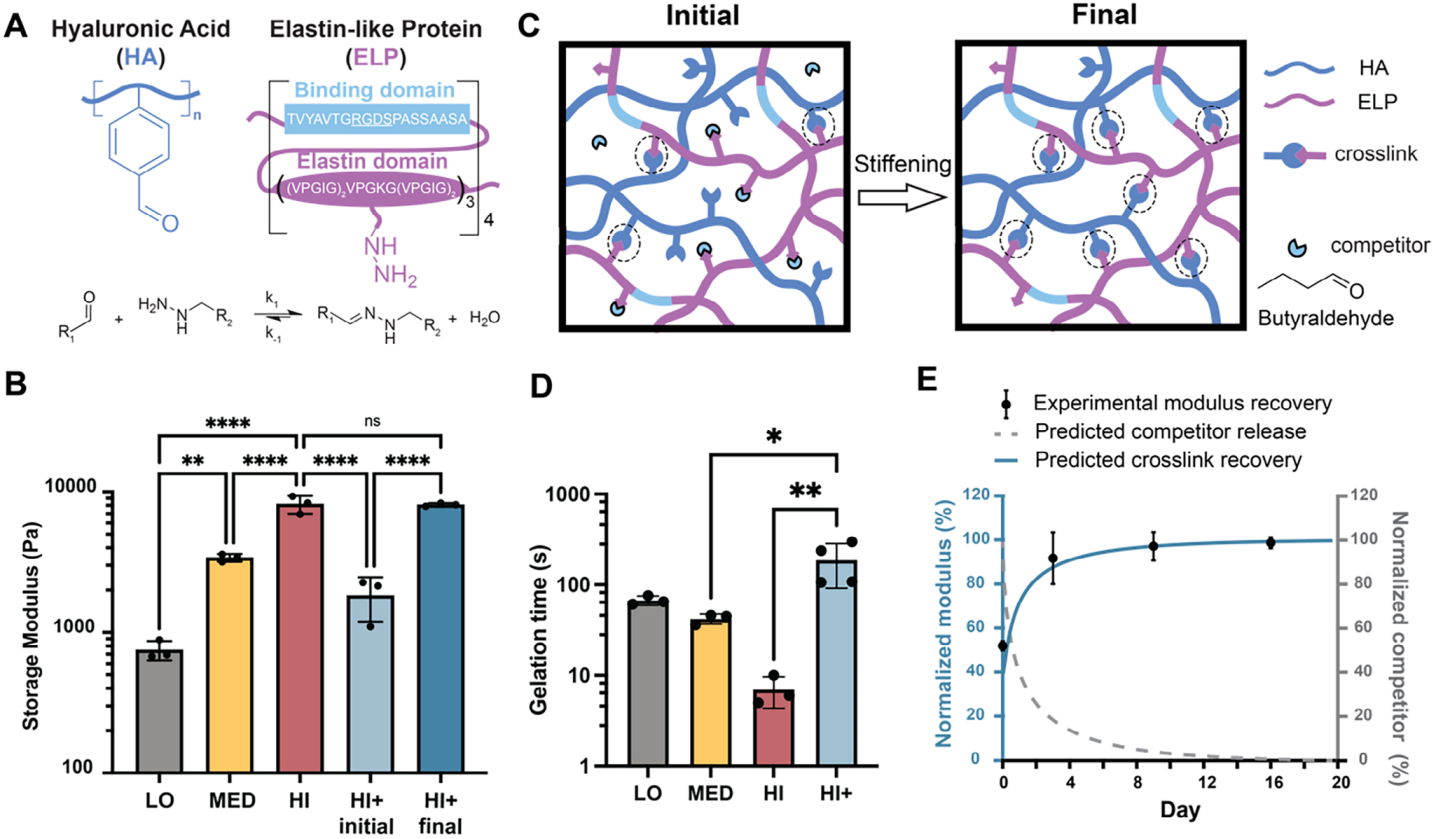
A library of hydrogels with different stiffness. A) Schematic of benzaldehyde‐modified hyaluronic acid and hydrazine‐modified elastin‐like protein (HELP), which can reversibly react. B) Storage modulus of HELP hydrogels with low stiffness (LO), medium stiffness (MED), high stiffness (HI), the initial state of HELP HI with competitors (HI+ initial), and the final state of HELP HI with competitors after diffusion (HI+ final). C) Schematic of the HELP hydrogel, with the reaction of aldehyde and hydrazine forming dynamic covalent crosslinks. The formation of crosslinks can be transiently hindered by including a butyraldehyde competitor that reversibly reacts with hydrazine groups (Initial) and can diffuse out of the hydrogel over time, resulting in a stiffer hydrogel (Final). Cross‐linking sites are circled with dotted lines. D) Gelation time of different HELP gels formulations. E) Theoretically predicted cumulative release of 10 × 10^−3^ m competitor over 20 d (gray dashed line), along with experimentally measured (black dots) and theoretically predicted (blue solid curve) modulus recovery over time. Predicted modulus recovery is assumed to be directly related to the theoretical percentage of hydrazone crosslinks formed. Data shown are mean ± sd of *N* = 3−4. Statistical significance was tested by one‐way ANOVA with Tukey's multiple comparisons testing: ns = no significant difference, * = *p* < 0.05, ** = *p* < 0.01, **** = *p* < 0.0001.

To investigate the effects of matrix stiffness on HO during NAFLD, we initially explored a previously reported family of tunable, engineered hydrogels consisting of hyaluronic acid (HA) and elastin‐like protein (ELP), termed HELP (**Figure** **1**A).^[^ ^48^, ^49^, ^50^ ^]^ HA is a common biopolymer found in liver ECM that increases during NAFLD progression.^[^ ^51^ ^]^ ELP is a recombinant, engineered protein composed of alternating cell‐adhesive domains for cell adhesion and repetitive elastin‐derived domains to provide structural support.^[^ ^52^ ^]^ Elastin is a common connective tissue component in normal liver tissue that also accumulates during liver fibrosis.^[^ ^53^ ^]^ Hydrogel formation is achieved by the crosslinking of benzaldehyde‐modified HA (HA‐BZA) and hydrazine‐modified ELP (ELP‐HYD) (Figure 1A; Figures S1 and S2, Supporting Information), to form dynamic covalent hydrazone bond crosslinks.^[^ ^48^ ^]^ Elastin‐like proteins are a type of intrinsically disordered protein that is highly dynamic.^[^ ^54^, ^55^ ^]^ Similarly, HA is a hydrophilic biopolymer that is expected to follow a dynamic, self‐avoiding random walk configuration in physiological buffer. Thus, when crosslinked together to form a hydrogel, the resulting HELP gels are completely amorphous and devoid of any microscale morphology, as previously demonstrated using Coherent anti‐Stokes Raman scattering microscopy.^[^ ^48^ ^]^

We first examined which cell‐adhesive ligands were sufficient to induce robust human hepatic spheroid growth in our minimal matrix. Based on previous reports that the integrin‐binding RGD peptide can successfully support hepatic cultures in other engineered materials,^[^ ^41^, ^42^ ^]^ we incorporated an extended RGD peptide sequence derived from fibronectin into our recombinant ELP. We formulated hydrogels with RGD concentrations of 1 or 2 × 10^−3^ m, while keeping the total ELP‐HYD concentration (2 wt%), HA‐BZA concentration (1 wt%), and matrix stiffness (*G*′ ≈ 3 kPa) constant. In addition, other reports have suggested that the incorporation of laminin or laminin‐derived peptides can improve hepatic culture growth.^[^ ^37^ ^]^ Thus, we also tested hydrogel with ELP variants that incorporated the laminin derived YIGSR or IKVAV peptides (at 1 × 10^−3^ m each) while maintaining a constant 1 × 10^−3^ m RGD concentration.^[^ ^52^ ^]^ Importantly, the concentration of ELP and HA were kept constant at 2 and 1 wt%, respectively. In addition, we examined the impact of incorporation full‐length mouse laminin‐111 (0.1 wt%) within our HELP formulation. Based on bright field microscopy on day 9, the HELP formulation with RGD and mouse laminin‐111 resulted in an increase in spheroids formed from single cells (Figure S3A,B, Supporting Information). Thus, all HELP gels used for the in vitro model throughout this study included these two components (RGD and laminin‐111).

Having identified the appropriate cell‐adhesive ligands to include in our minimal hepatic matrix, we next sought to develop HELP gels with a range of stiffness to mimic healthy (*G*′ ≈ 0.8 kPa), early onset (*G′* ≈ 3 kPa), and advanced NASH (*G*′ ≈ 6 kPa).^[^ ^56^, ^57^ ^]^ Similar to HA and elastin, fibronectin is also upregulated during NAFLD, with increasing expression correlated with fibrosis.^[^ ^58^, ^59^ ^]^ Thus, we tuned the concentrations of our chemically modified biopolymers to form HELP gels with LO, MED, and HI stiffness (Figure 1B). These gels all included laminin‐111 (0.1 wt%) and the RGD‐containing ELP variant, with ELP‐HYD/HA‐BZA concentrations of 1:1, 2:1, and 4:2 wt%, respectively. While robust HO formation was observed in the HELP LO and MED conditions, the HELP HI gels resulted in irreproducible, low levels of organoid formation (Figure S4, Supporting Information). This observation is consistent with published results for mouse liver organoids, where higher stiffness matrices resulted in less efficient organoid formation.^[^ ^42^ ^]^ In addition, the high biopolymer concentrations in HELP HI gels caused rapid gelation (<10 s), frequently leading to inhomogeneous mixing of cells and irreproducible cultures (Figure S4, Supporting Information).

To overcome these challenges with the HELP HI gels, a small molecule competitor, butyraldehyde (10 × 10^−3^ m), was added to the HELP HI formulation to reversibly disrupt the hydrazone crosslinks (Figure 1C).^[^ ^60^ ^]^ We term this gel formulation HELP HI+. As expected, this disruption of crosslink formation resulted in a hydrogel with an initially decreased stiffness (Figure 1B, HELP HI+ initial, *G′* ≈ 1.5 kPa). When the gels are immersed in competitor‐free media, the competitors can diffuse away over time to allow the formation of additional hydrazone crosslinks, resulting in a final gel stiffness similar to HELP HI formulations without competitor (Figure 1B, HELP HI+ final, *G*′ ≈ 6 kPa). Final gel stiffness measurements were taken on day 16 to match the time period of hepatic organoid culture for these studies. Moreover, adding the competitor increased the gelation time from <10 s for HELP HI to >100 s for HELP HI+ (Figure 1D). By using the 1D reaction‐diffusion model we developed in previously published work,^[^ ^60^ ^]^ we theoretically predicted the competitor release and crosslink recovery over time, which showed good agreement with the experimentally measured storage modulus over 16 d (Figure 1E). We confirmed that the competitors fully diffused out of the gels within 16 d, and the final gel stiffness had recovered to its expected value (≈6 kPa).

### 2.2 Liver‐Mimetic Hydrogels Support Hepatic Organoid Growth

**Figure 2:**
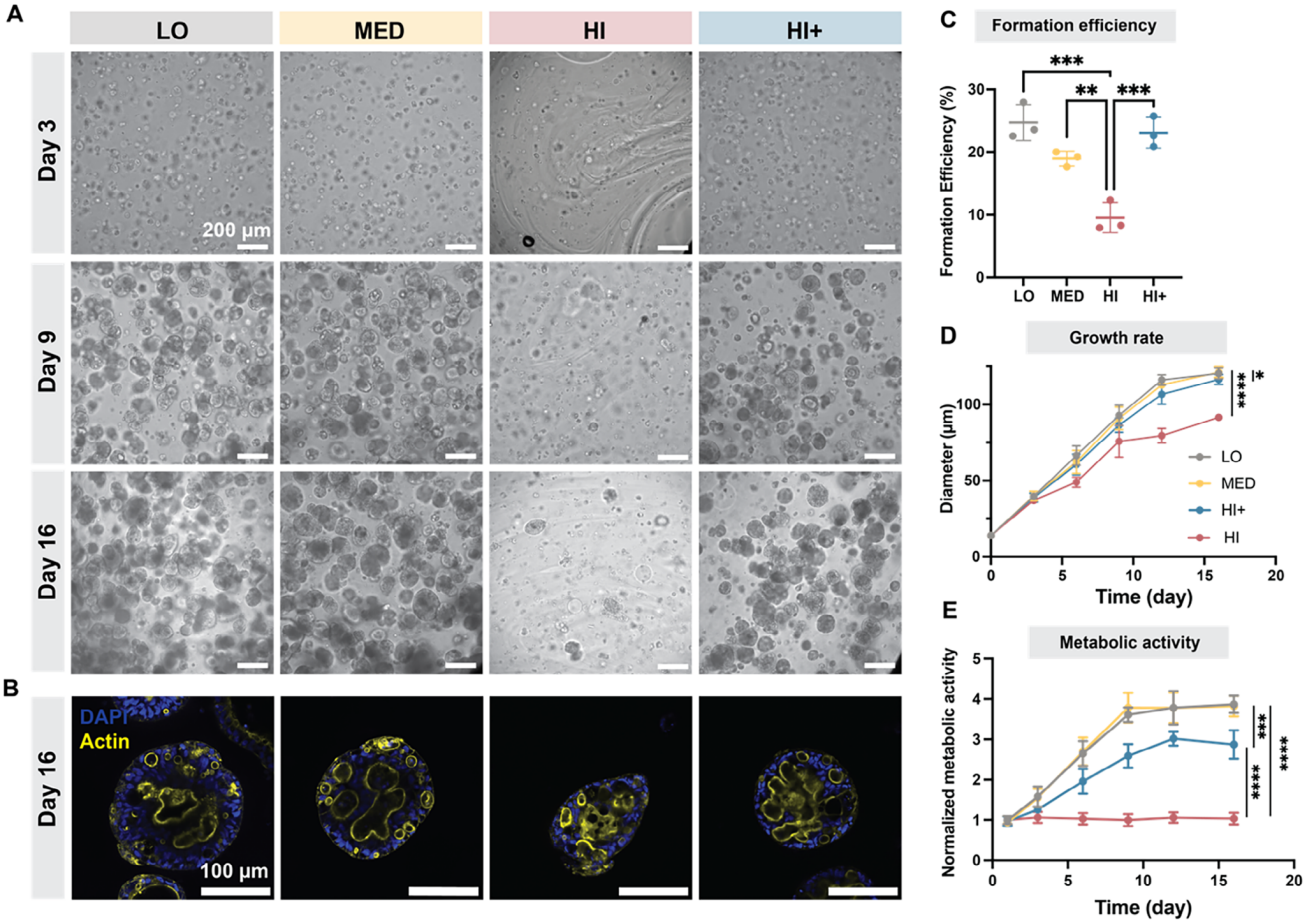
HO growth in four different HELP hydrogel conditions. A) Brightfield images of HOs cultured in all HELP formulations at days 3, 9, and 16. B) Confocal fluorescence of HO morphology at day 16 in different HELP gels. C) Formation efficiency of spheroids on day 3. D) Growth rate of encapsulated HOs through 16 d from single cells, as measured by the average organoid diameter. E) Metabolic activity of encapsulated HOs through 16 d. Data shown are mean ± sd of *n* = 3 replicate cultures. Statistical significance was tested by one‐way ANOVA with Tukey's multiple comparisons testing: ns = no significant difference, * = *p* < 0.05, ** = *p* < 0.01, *** = *p* < 0.001, **** = *p* < 0.0001.

We then examined HOs formation and growth within all four HELP gel formulations over time. As expected, on day 0 and day 3, we observed more homogenous gels and more evenly distributed cells in HELP HI+ compared to HELP HI (**Figure** **2**A; Figure S5A, Supporting Information), presumably due to the increased gelation time, which allows for more complete mixing. Y‐27632 is a Rho Kinase (ROCK) inhibitor that enhances the survival of human stem cells after disassociation into single cells^[^ ^61^ ^]^ and is commonly added to culture medium after organoid passaging to increase cloning efficiency.^[^ ^38^, ^42^ ^]^ We observed that addition of Y27632 for the first 9 d of culture (and removed in the following days) greatly enhanced spheroid formation and growth (Figure S5B, Supporting Information). When following this Y27632 protocol for all four gel formulations, spheroid formation efficiency in HELP HI+ was qualitatively comparable to the HELP LO and MED formulations (Figure 2A).

Importantly, the presence of the competitor in HELP HI+ did not lead to an aberrant morphology compared to other conditions (Figure 2B). In addition, the presence of competitor resulted in organoid formation efficiency (≈20%, Figure 2C) and organoid growth rate (Figure 2D) for HI+ that was significantly higher than that for HI and comparable to LO and MED. Cellular metabolic activity in HI+ was lower than LO and MED, although HI+ did result in a statistically significant approximately threefold increase in metabolic activity compared to HI (Figure 2E). Taken together, these data suggest that the dynamic stiffening matrix resulted in a marked improvement in organoid culture reproducibility, enabling the efficient formation and growth of HOs within stiff matrices.

**Figure 3:**
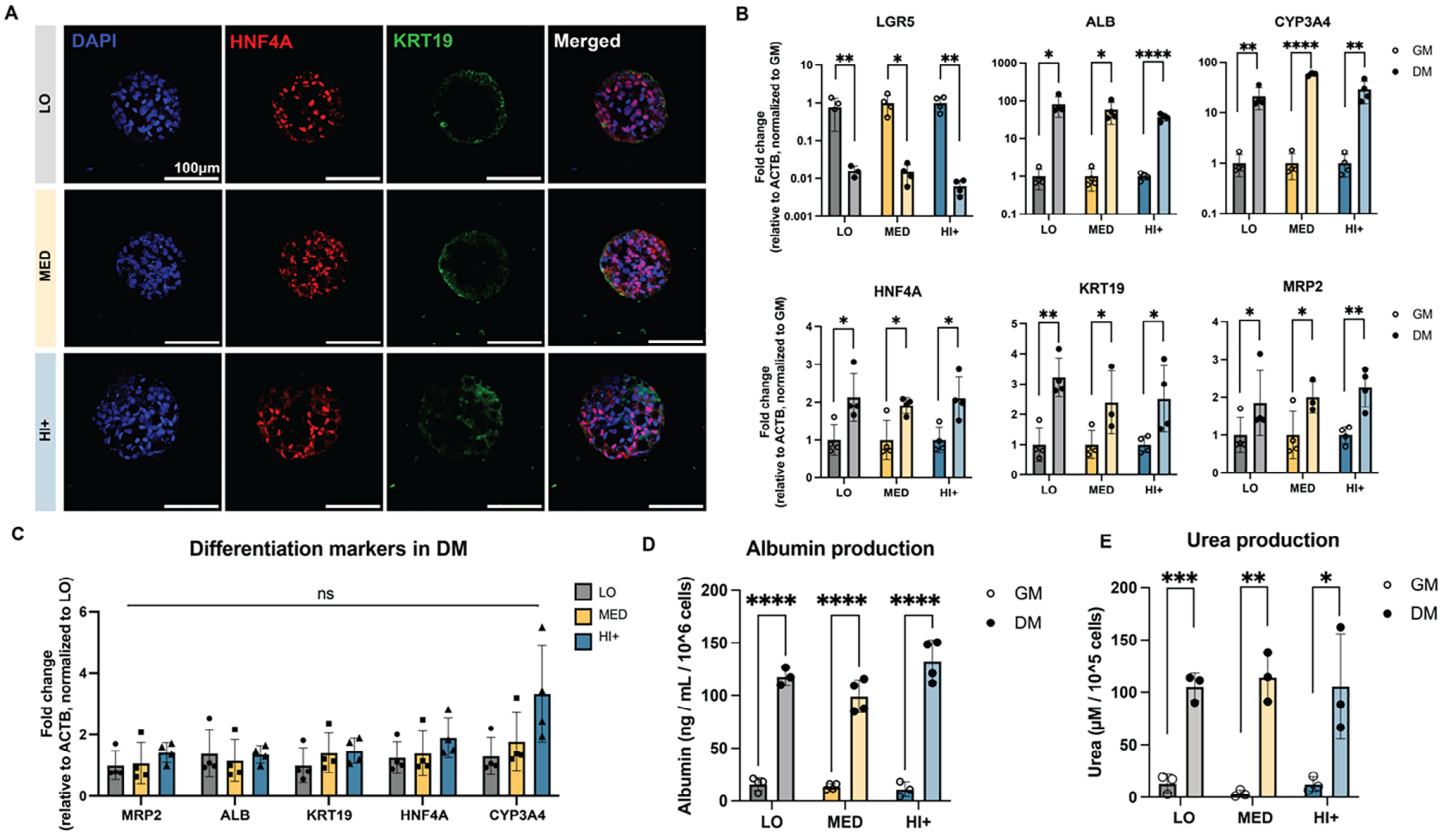
Phenotypic analysis of HO differentiation. A) Representative confocal fluorescence images of differentiated HOs on day 16 in different HELP matrices for hepatocyte (HNF4A, red) and cholangiocyte (KRT19, green) markers with nuclear DAPI counterstain (blue). B) qRT‐PCR analysis of gene expression of HO stem (*LGR5*) and differentiation markers (*ALB, CYP3A4, HNF4A, KRT19, MRP2*) for organoids grown in growth media (GM) and differentiation media (DM). ΔΔCT relative to β‐actin (*ACTB*) was normalized to HOs grown in GM in each stiffness condition. C) Gene expression of HO differentiation markers was analyzed by RT‐qPCR in differentiation media (DM). ΔΔCT relative to β‐actin (*ACTB*) was normalized to HOs grown in HELP LO. D) Albumin production was quantified from the supernatant of organoids grown within the three matrices in GM and DM and normalized to total cell number. E) Urea production was quantified from the supernatant of organoids grown within the three matrices in GM and DM and normalized to total cell number. Data shown are mean ± sd of *n* = 3–4 replicate cultures. Statistical significance was tested by one‐way ANOVA (panel C) with Tukey's multiple comparisons testing or unpaired two tailed Student's *t* test (panels B, D, E): * = *p* < 0.05, ** = *p* < 0.01, *** = *p* < 0.001, **** = *p* < 0.0001; *n* = 3–4 replicate cultures.

We next characterized the phenotype of hepatic organoids grown within the LO, MED, and HI+ matrices. In growth medium, hepatic spheroids cultured in these three matrices showed similar morphology on day 9, with positive staining for cell proliferation (Ki67) and the cell membrane protein E‐cadherin (Figure S6, Supporting Information). On day 9, we exchanged the growth medium with differentiation medium for 7 further days of culture (i.e., total of 16 d). Histology (hematoxylin and eosin staining) showed that HOs cultured in LO, MED, and HI+ stiffness conditions had similar size and mature morphology after the full 16 d (Figure S7, Supporting Information), similar to previously reported liver organoids.^[^ ^44^, ^62^ ^]^ Differentiated organoids in all matrices expressed hepatocyte (*HNF4A*) and cholangiocyte (*KRT19*) markers (**Figure** **3**A). Compared to organoids cultured in GM, organoids in DM showed significantly decreased stem cell marker LGR5 expression and exhibited significantly higher transcription levels of mature hepatocyte markers including multi‐drug resistance protein 2 (*MRP2*), albumin (*ALB*), keratin 19 (*KRT19*), hepatocyte nuclear factor 4 alpha (*HNF4A*), and cytochrome P450 3A4 (*CYP3A4*) (Figure 3B), with comparable expression in all matrix conditions (Figure 3C). Quantification of albumin secretion (Figure 3D) and urea production (Figure 3E) was found to be significantly higher for differentiated organoids (DM) compared to undifferentiated control cultures (GM), with comparable levels of albumin and urea produced across the three different matrix conditions. We further conducted a rhodamine 123 transport assay to evaluate the activity of multidrug resistant protein 1 (*MRP1*), an apical p‐glycoprotein efflux transporter that mediates drug secretion into the lumen of the organoids.^[^ ^63^ ^]^ When exposed to rhodamine 123, organoids accumulated fluorescence inside their lumens (Figure S8, Supporting Information). In contrast, organoids pretreated with the MRP1 inhibitor verapamil showed an accumulation of rhodamine 123 only in the cell cytoplasm, with no accumulation in the lumens (Figure S8, Supporting Information), indicating MRP1‐specific transport. Thus, across our three stiffness conditions at day 16, differentiated HOs displayed gene expression levels, protein expression levels, and transport function indicative of successful hepatic differentiation and maturation.

### 2.3 Increased Lipid Droplet Accumulation is Observed in High Stiffness Matrices

**Figure 4:**
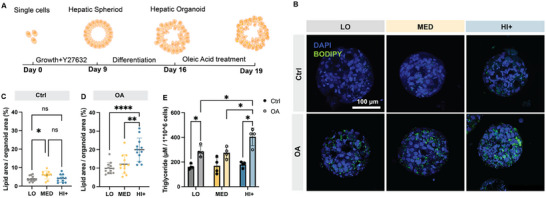
Lipid droplet accumulation post oleic acid (OA) treatment. A) Schematic of the HO culture protocol within HELP matrices. HOs were grown from single cells to form differentiated organoids over 16 days, then treated with 500 × 10^−6^ m OA for another three days. B) Representative fluorescent image max projections of HOs stained with DAPI (nuclei) and BODIPY (lipid droplets) in the three matrix conditions, either treated with 0 × 10^−6^ m OA (Ctrl groups) or 500 × 10^−6^ m OA (OA groups). C,D) Image quantification of lipid area percentage in Ctrl (C) and OA (D) groups. Data shown are mean ± sd of *N* = 12, *n* = 3–4 replicate cultures. Statistical significance was tested by one‐way ANOVA with Tukey's multiple comparisons testing: ns = no significant difference, * = *p* < 0.05, ** = *p* < 0.01, **** = *p* < 0.0001. E) Triglyceride accumulation normalized to cell number in all conditions. Data shown are mean ± sd of *n* = 4 replicate cultures. Two‐way ANOVA with Tukey's multiple comparisons testing; * = *p* < 0.05.

Dietary habits such as high caloric intake and high carbohydrate/fat consumption are key risks associated with NAFLD.^[^ ^64^ ^]^ To develop steatosis models that capture this diet‐related risk factor, we exposed differentiated HOs in the three HELP matrices to oleic acid (OA, 500 × 10^−6^ m) for 3 d (**Figure** **4**A). OA is one of the most abundant FFA in human plasma.^[^ ^65^ ^]^ Without OA treatment, organoids showed relatively low amounts of lipid droplet staining (less than 10% of the organoid area at day 19, Figure 4B,C). In contrast, with OA treatment, there was an increase in lipid droplet accumulation across all matrix conditions, creating a model of diet‐related steatosis in NAFLD (Figure 4B,D). Notably, we observed significantly higher lipid droplet accumulation in HI+ compared to LO and MED matrices (Figure 4D). As OA is commonly converted and stored as triglycerides in liver cells,^[^ ^66^ ^]^ we quantified triglycerides on a per cell basis with and without OA treatment. HOs within the HI+ matrix had statistically higher triglyceride concentration compared to LO and MED (Figure 4E). These results suggested that matrix stiffness may promote OA uptake and lipid droplet accumulation and encouraged us to further characterize the intracellular lipids.

**Figure 5:**
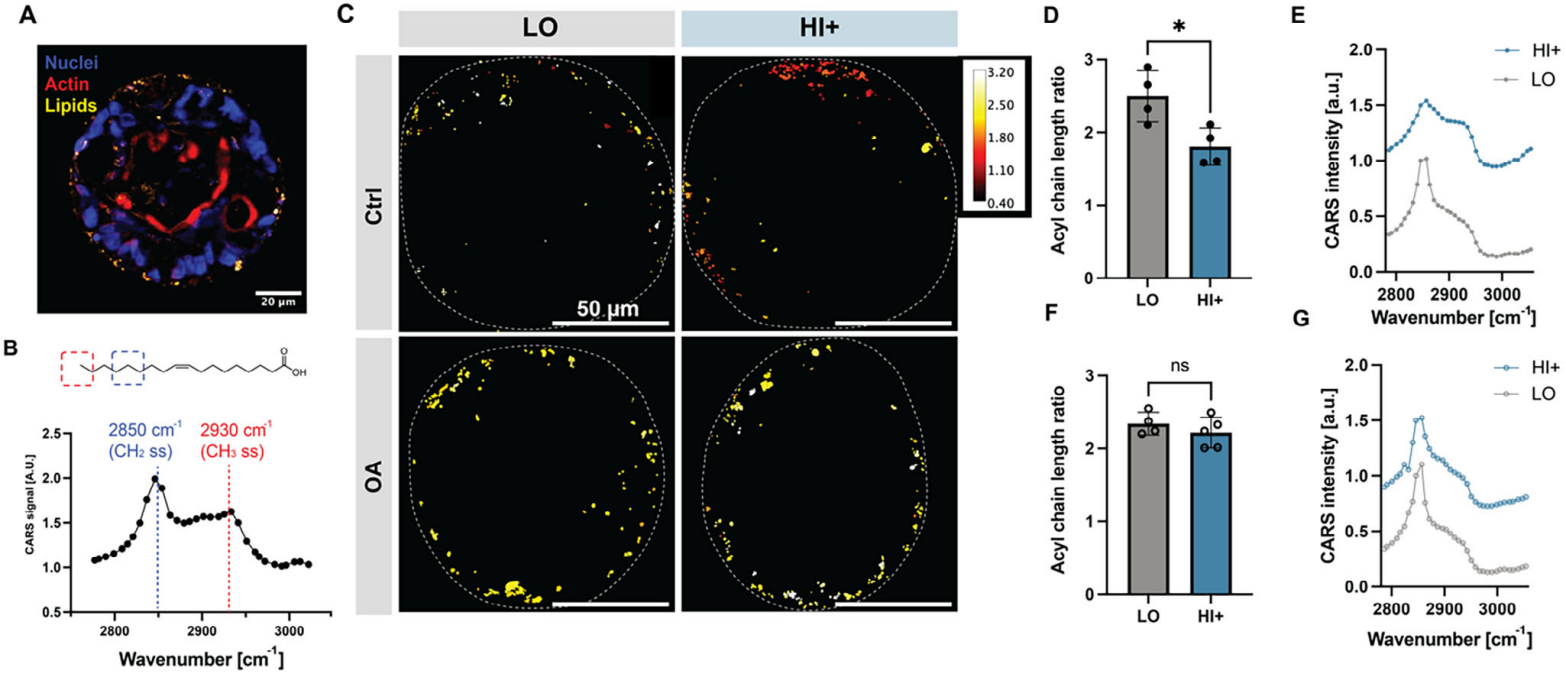
Lipid analysis using coherent anti‐Stokes Raman scattering (CARS) microscopy. A) Representative multiplexed CARS/confocal fluorescence image of a HELP‐encapsulated HO stained with DAPI (nuclei) and phalloidin (actin) and with lipid distribution captured by CARS microscopy. B) CARS spectrum of oleic acid demonstrating the characteristic peaks for CH_2_ and CH_3_ groups. 2850 cm^−1^ is assigned to the CH_2_ symmetric stretching vibration, and 2930 cm^−1^ is assigned to the CH_3_ symmetric stretching vibration. Hence, the peak intensity ratio I_2850_ / I_2930_ is defined as a parameter related to the lipid acyl chain length. C) Representative maps of the CARS intensity ratio for lipid droplets in HELP‐encapsulated HOs with or without OA treatment in LO and HI+ matrices. Droplets are color‐coded from dark red to white to represent lower to higher I_2850_ / I_2930_ ratios, respectively. D,E) Average I_2850_ / I_2930_ ratios for HELP‐encapsulated HOs in (D) control groups without OA treatment and (E) OA‐treated groups. F,G) Representative CARS spectra for lipids in HOs in (F) control groups and (G) OA‐treated groups. Data shown are mean ± sd of *N* = 4–5 organoids from *n* = 3 replicate cultures. Unpaired two tailed Student's *t* test; ns = no significant difference, * = *p* < 0.05.

To evaluate if potential changes in lipid composition are occurring in different stiffness matrices, we used coherent anti‐Stokes Raman scattering (CARS) microscopy to visualize and characterize the composition of individual lipid droplets. CARS microscopy is a nonlinear optical imaging method that leverages the inherent molecular vibrations of structures to visualize them without the need for added labels or stains.^[^ ^67^ ^]^ Due to a quadratic signal dependence on the number density of the probed vibrations, CARS microscopy of CH_2_ symmetric stretching vibrations visualizes lipid droplets with excellent contrast as they contain a high concentration of methylene groups, which allows their separation from other biomolecules (e.g., proteins or DNA) with different molecular bond ratios.^[^ ^68^ ^]^ In addition to quantitative spatial information, CARS can also provide qualitative chemical information about the lipid droplets in intact cells by evaluating their vibrational spectra.^[^ ^69^ ^]^ By multiplexing CARS microscopy with fluorescence confocal microscopy, we were able to image the lipid droplets (CARS), nuclei (fluorescence), and actin morphology (fluorescence) within the same sample (**Figure** **5**A). As a first analysis, we quantified the total lipid droplet volume per organoid, complemented by the average lipid droplet size in HOs across all three conditions (Figure S9A, Supporting Information). The stiffer HI+ condition showed larger individual lipid droplet and larger total lipid droplet volumes per organoid (Figure S9B,C, Supporting Information), which was in good agreement with the observations made on the BODIPY stained organoids (Figure S9B–E, Supporting Information). To further evaluate whether there are lipid composition differences between organoids cultured in LO and HI+ matrices, spectral series of CARS images were collected and analyzed. While the signal at 2850 cm^−1^ is assigned to CH_2_ symmetric stretching vibrations, 2930 cm^−1^ can be assigned to CH_3_ symmetric stretching (Figure 5B).^[^ ^69^ ^]^ Thus, the ratio of the CARS intensity at these two wavenumbers (I_2850_ / I_2930_) was used to define a parameter that relates to the acyl chain length of lipids (since CH_2_ groups are found along the length of the acyl chain, while a single CH_3_ group is found only at the end of the chain).^[^ ^69^ ^]^ Interestingly, the CARS spectra suggest that lipid droplet accumulated in HOs contain lipids with shorter acyl chains in the stiffer HI+ condition compared to the LO condition prior to OA treatment (Figure 5C). Quantification of the CARS ratio showed a significantly lower normalized intensity ratio for the HI+ condition (I_2850_ / I_2930_ ratio ≈ 1.8, suggesting relatively shorter acyl chains) to the LO condition (I_2850_ / I_2930_ ratio ≈ 2.4) (Figure 5D,E). This corresponds well to results obtained from a lipidomic study of human liver biopsies, showing increased amounts of short and saturated fatty acyl chain‐containing species in fibrotic NASH relative to steatosis.^[^ ^70^ ^]^ Taken together, these data suggested that increased matrix stiffness may result in altered regulation of lipid metabolism pathways, giving rise to different intrinsic lipid compositions. In contrast, the acyl chain ratio obtained from the spectral CARS images was statistically similar for the two matrices post‐OA treatment (Figure 5C,F,G). This suggests that the uptake and processing of exogenous fatty acids dominate the system and prevent detection of endogenous changes in lipid composition. These results prompted us to explore gene expression of some of the protein pathways involved in lipid metabolism.

### 2.4 HOs Display Increased Expression of Fibrosis and Lipid Metabolism Markers in High Stiffness Matrices

**Figure 6:**
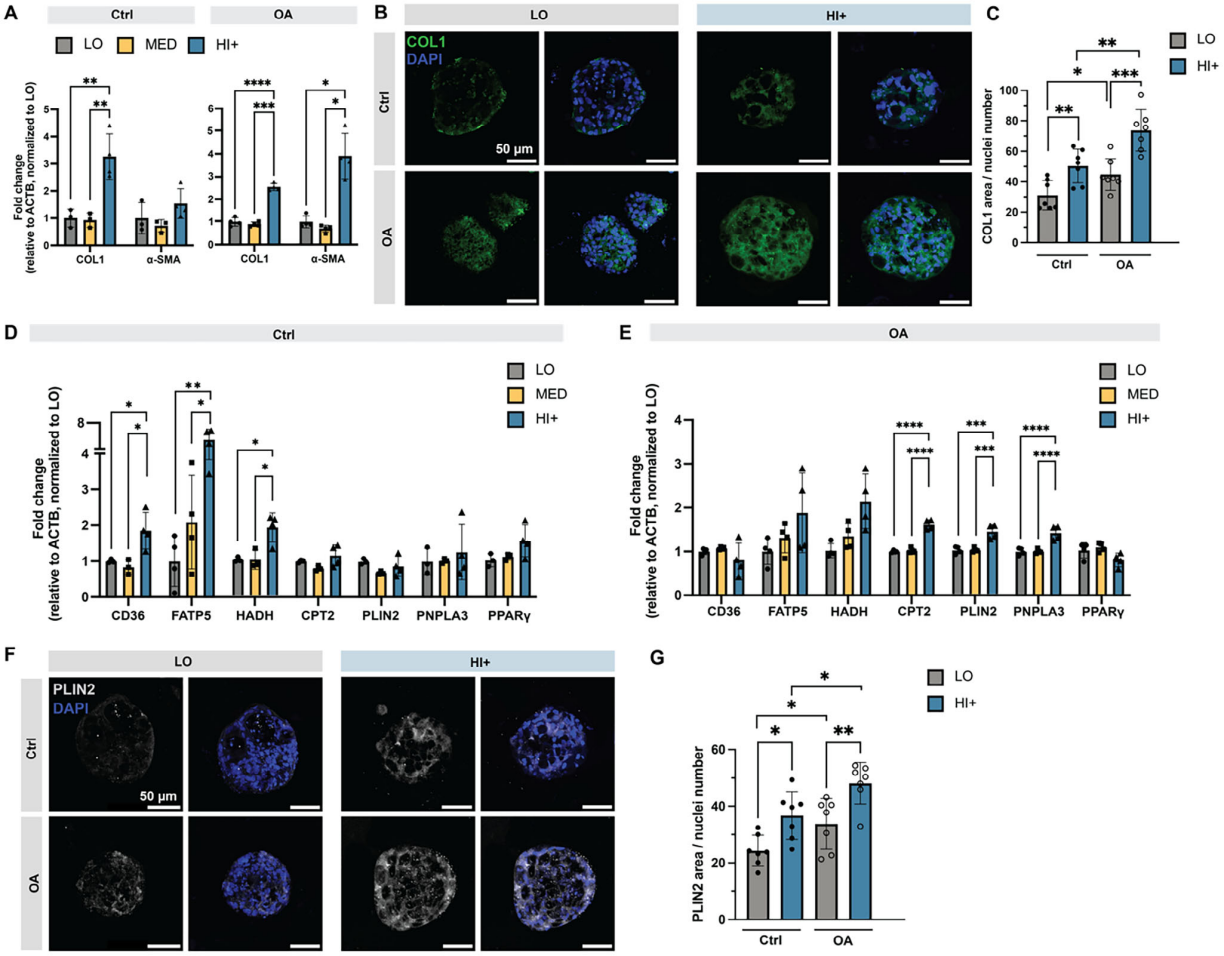
Expression of fibrosis markers and lipid metabolism markers in different stiffness matrices. A) mRNA expression of fibrosis genes in HOs in different stiffness matrices with 0 × 10^−6^ m OA (Ctrl) or 500 × 10^−6^ m OA (OA) treatment. ΔΔ*C*_T_ relative to β‐actin (*ACTB*) was normalized to LO. B) Representative immunofluorescence images of *COL1* immunostaining in HOs cultured in LO and HI+ matrices with 0 or 500 × 10^−6^ m OA treatment. C) Quantification of *COL1*. D,E) mRNA expression of lipid metabolism genes in HOs in different stiffness matrices with D) 0 × 10^−6^ m OA or E) 500 × 10^−6^ m OA treatment. ΔΔ*C*_T_ relative to β‐actin (*ACTB*) was normalized to LO. F) Representative immunofluorescence images of *PLIN2* immunostaining in HOs cultured in LO and HI+ matrices with 0 or 500 × 10^−6^ m OA treatment. G) Quantification of *PLIN2*. Data shown are mean ± sd; Statistical significance was tested by one‐way (panels A, D, E) or two‐way (panels C, G) ANOVA with Tukey's multiple comparisons testing: * = *p* < 0.05, ** = *p* < 0.01, *** = *p* < 0.001, **** = *p* < 0.0001; *n* = 3–4 replicate cultures (panels A, D, E); *n* = 3–4 replicate cultures with *N* = 7–8 organoids (panels C, G).

To characterize the gene expression of the encapsulated HOs at the mRNA level, we performed qRT‐PCR on cells cultured within all three gel formulations in nontreated (Ctrl) and OA‐treated (OA) groups. Previous reports have shown that liver fibrosis increases during NAFLD progression, both in in vivo and in vitro models.^[^ ^9^, ^10^ ^]^ Here, we found that organoids upregulated expression of collagen 1 (*COL1*) in HI+ conditions compared to lower stiffness matrices even when there was no OA stimulation. (**Figure** **6**A, ctrl). Post OA treatment, *COL1* remained upregulated in the stiffest HI+ matrices, and α‐smooth muscle actin (α‐SMA) upregulation was also observed in HI+ conditions. (Figure 6B, OA). When each matrix stiffness condition was considered individually, both α‐SMA and *COL1* were significantly up‐regulated upon OA stimulation (Figure S10, Supporting Information). These data suggest that matrix stiffness and OA treatment both were effective at driving the cultures towards increased fibrosis in this HO model. To confirm this gene expression data at the protein level, we performed immunostaining for *COL1*. Consistent with the qRT‐PCR results, both increased matrix stiffness and treatment with OA was found to statistically increase *COL1* immunostaining (Figure 6B,C). Importantly, this level of COL1 expression in our model is insufficient to induce further fibrosis or tissue stiffening, as we did not observe increased stiffness of matrices after 16 d of cell culture (Figure S11, Supporting Information).

NAFLD progression is also known to result in lipid metabolic dysfunction;^[^ ^71^, ^72^ ^]^ thus, we next explored the impact of OA and matrix stiffness on several genes involved in fatty acid transport and lipid metabolism. In control groups, i.e., without exogenous OA treatment, *CD36* (a fatty acid transporter) and *FATP5* (fatty acid transport protein 5) had higher expression in HI+ compared to LO and MED matrices (Figure 6D). HOs in HI+ matrices also showed increased expression of hydroxyacyl‐CoA dehydrogenase (*HADH*), an enzyme that catalyzes the beta oxidation in mitochondria, whereas other lipid metabolism‐related genes had comparable expression across different matrices. Since long chain fatty acids are broken down during beta oxidation in mitochondria, this result may explain the shorter acyl chain length suggested by the CARS analysis of lipid droplets in the HI+ condition (Figure 5C–E).

Post OA treatment, HOs in HI+ matrices displayed significantly higher expression of several lipid metabolism related genes, including droplet coating protein perilipin 2 (*PLIN2*), carnitine palmitoyltransferase 2 (*CPT2*), and patatin‐like phospholipase domain‐containing protein 3 (*PNPLA3*). Furthermore, while not statistically significant, expression levels of *HADH* and *FATP5* were trending upward in the HI+ stiffer matrices post‐OA treatment (Figure 6E). In patient data, *PLIN2*, *CPT2*, and *PNPLA3* upregulation is strongly associated with NFALD.^[^ ^73^ ^]^ The enzyme *CPT2* plays a key role in transporting long‐chain fatty acids into the mitochondria where they can be broken down by beta‐oxidation.^[^ ^72^, ^74^ ^]^ *PLIN2* is widely expressed in the liver and plays an important role in lipid droplet formation and accumulation.^[^ ^75^ ^]^ *PNPLA3* encodes for an enzyme that helps to regulate fat metabolism and has been shown to be strongly associated with NAFLD.^[^ ^73^, ^76^ ^]^ When comparing gene expression levels pre‐ and post‐OA treatment for each individual matrix stiffness, *PLIN2* and *CPT2* increased significantly with exposure to FFA. (Figure S10, Supporting Information) In contrast, peroxisome proliferator‐activated receptor (*PPAR*γ, a transcription factor related to lipid metabolism and inflammation) was relatively stable pre‐ and post‐OA treatment and across all matrix stiffness conditions (Figure 6E; Figure S10, Supporting Information).

These results are in good agreement with previous findings that FFA treatment can drive organoids towards more steatosis and metabolic dysfunction.^[^ ^30^, ^77^ ^]^ Moreover, these data suggest that ECM stiffening, such as that which occurs during NAFLD progression, can impact lipid metabolism of hepatic organoids. Immunostaining for protein expression revealed that increased matrix stiffness or exposure to OA could significantly increase *PLIN2* levels (Figure 6F,G). *PLIN2* expression was highest in the stiff HI+ matrix with OA treatment, suggesting the potential for greater lipid storage in this condition, which is consistent with the previous lipid droplet measurements (Figure 5G). In addition to alterations in lipid metabolism, NAFLD patients also have decreased ability to metabolize drugs, toxins, and other foreign organic molecules,^[^ ^78^ ^]^ as characterized by a decrease in the enzyme cytochrome P450 3A4 (*CYP3A4*).^[^ ^79^ ^]^ Accordingly, we observed a significant down‐regulation of *CYP3A4* in all stiffness conditions post‐OA treatment at both the gene expression (Figure S12A, Supporting Information) and protein expression levels (Figure S12B,C, Supporting Information). Overall, these results indicate that our model mimics many of the transcriptional and protein changes associated with clinical pathological progression of NAFLD, including markers for increased expression of matrix proteins, abnormal lipid metabolism, and impaired drug metabolism.

### 2.5 Inhibitor‐Induced Reduction of Steatosis

As there are very few FDA‐approved drugs for NAFLD, we next explored the potential inhibitors that can be used to reduce markers of steatosis within our HO model. Our gene expression data suggested that fatty acid transporter *FATP5* was up regulated in stiffer HI+ stiffness matrices (Figure 6D). Secondary bile acids have the potential to block fatty acid transporter function in liver cells.^[^ ^80^ ^]^ Specifically, deoxycholic acid (DCA) has been shown to effectively inhibit *FATP5* in mice to reduce diet‐induced hepatic FFA uptake and lipid accumulation.^[^ ^80^ ^]^ Here, we added 20 × 10^−6^ m DCA during the 3‐d OA treatment and quantified the lipid droplet accumulation in both LO and HI+ conditions. As expected, both groups showed significant decrease of lipid droplet accumulation compared to OA‐treatment alone (Figure S13, Supporting Information). Interestingly, even with DCA treatment, we still observed an indication of different in lipid droplet accumulation between the LO and HI+ stiffness conditions, which motivated us to explore mechano‐signaling inhibitors as a potential treatment.

**Figure 7:**
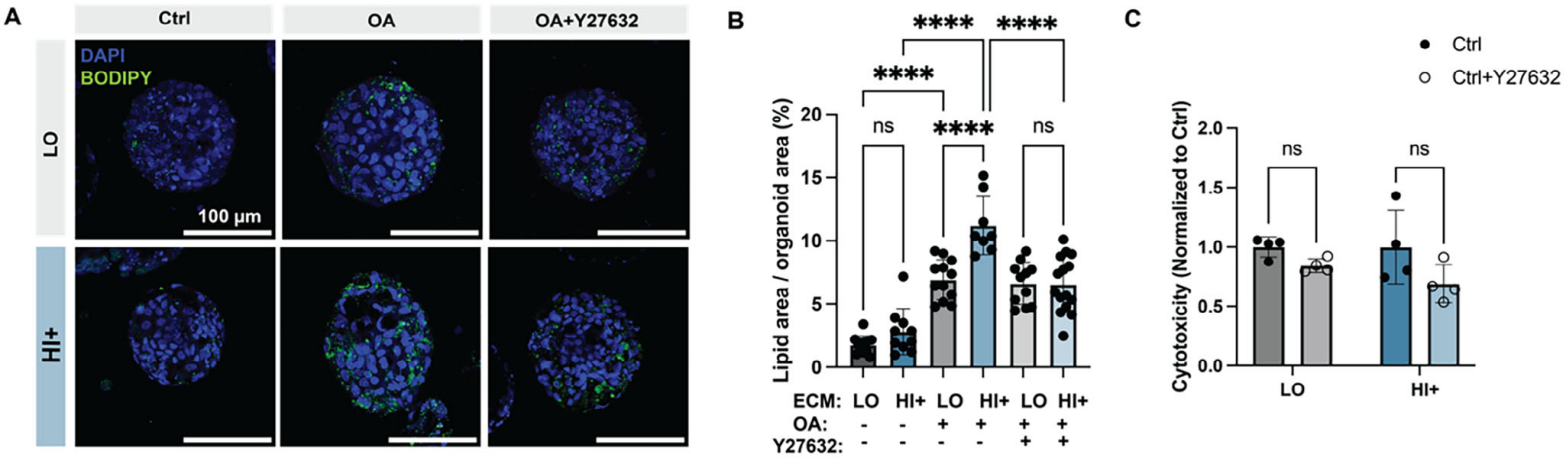
Mechanosignaling inhibition with Y27632 in LO and HI+ matrices. A) Representative fluorescent images of HOs stained with DAPI (nuclei) and BODIPY (lipids) in LO and HI+ matrix conditions, treated with 0 × 10^−6^ m OA (Ctrl groups), 500 × 10^−6^ m OA, or 500 × 10^−6^ m OA, and 20 × 10^−6^ m Y27632. B) Quantification of lipid area as percentage of organoid area for each condition. C) Lactate dehydrogenase (LDH) cytotoxicity assay in LO and HI+ matrices with 0 or 20 × 10^−6^ m Y27632, normalized to 0 × 10^−6^ m control. Data shown are mean ± sd; Statistical significance was tested by one‐way ANOVA (panel B) with Tukey's multiple comparisons testing or unpaired two tailed Student's *t* test (panel C): * = *p* < 0.05, ** = *p* < 0.01, *** = *p* < 0.001, **** = *p* < 0.0001; *n* = 3–4 replicate cultures with *N* = 8–12 organoids (panel B); *N* = 3–4 replicate cultures (panel C).

Cells sense their surrounding matrix stiffness via several mechano‐signaling pathways. Rho/ROCK is a canonical pathway through which mechano‐stimuli (e.g., matrix stiffness) is converted into biochemical signals within the cells.^[^ ^81^ ^]^ Rho/ROCK regulates remodeling of the actin cytoskeleton to generate intracellular tension that leads to activation of metabolic functions, gene expression, cell polarization, cytoskeletal organization, proliferation, and differentiation.^[^ ^82^ ^]^ ROCK1 is up‐regulated in some NAFLD models, and pharmacological blockade of ROCK is suggested to prevent the progression of liver disease in these preclinical models.^[^ ^83^, ^84^ ^]^ Here, we inhibited the Rho/ROCK pathway using the antagonist Y27632 (20 × 10^−6^ m) during OA treatment. Interestingly, Y27632 treatment significantly reduced lipid droplet accumulation in the HI+ condition to be similar to that in the LO condition (**Figure** **7**A,B). Importantly, Y27632 treatment did not induce any cytotoxicity at the concentrations we applied (Figure 7C) and did not interfere with lipid droplet accumulation in the LO condition (Figure 7A,B). Together, these data suggest that the ROCK mechano‐signaling pathway is necessary for the observed increase in lipid droplet accumulation in stiffer matrices in our HO model, encouraging future work to further study the ROCK pathway as a potential mechano‐therapy to inhibit NAFLD progression.

## 3 Conclusion

Altogether, our results revealed that matrix stiffness influences hepatic organoid lipid droplet accumulation and lipid metabolism in a 3D microenvironment. We developed a family of HELP gels with different stiffness that mimicked distinct stages during fatty liver disease progression. Using small molecule competitors, we developed a dynamic stiffening hydrogel, which allowed us to achieve reproducible HO cultures even in stiff matrices (≈6 kPa). HOs showed comparable formation efficiency and growth rate in all stiffness conditions, with reproducible emergence of internal lumens and expression of liver differentiation markers. When treating HOs with OA, we observed significantly more lipid droplet accumulation in higher stiffness matrices. We further demonstrated that HOs showed higher expression of fibrosis and lipid metabolism markers in higher stiffness matrices. CARS analysis suggested that the accumulated lipid droplets in higher stiffness matrices had shorter acyl chain lengths, which would be consistent with increased beta oxidation. Finally, we showed that the stiffness‐induced steatosis in our HO model was prevented by treatment with a ROCK‐inhibitor (Y27632), encouraging further study of potential mechano‐therapeutics for NAFLD treatment.

Currently, there is only one FDA‐approved treatment for NAFLD patients,^[^ ^5^, ^6^ ^]^ and the development of NAFLD in vitro models for drug screening could significantly speed up the drug discovery pipeline.^[^ ^27^, ^33^, ^85^ ^]^ Compared to organoids cultured in Matrigel or suspension, which can suffer from batch‐to‐batch irreproducibility,^[^ ^35^, ^36^ ^]^ our platform uses a designed HELP hydrogel that offers a reproducible platform for HO culture and future screening of potential drugs for NAFLD treatment. Importantly, the ability to systematically tune the biochemical and mechanical properties of HELP hydrogels will enable further mechanistic studies to explore how changes in the matrix microenvironment during NAFLD progression impact lipid metabolic dysfunction. In future work, we can apply single or combined drug treatments to our model and conduct more systematic analyses of lipid accumulation, fibrosis marker expression, and metabolic function in hepatic organoids to comprehensively evaluate drug efficacy and mechanisms of action.

Our current HO model is well‐suited for mechanistic studies to dissect hepatocyte cell signaling pathways and lipid metabolism in response to matrix mechanics without confounding signals from other cell types. This simplified model does not include other cell types present in the liver, such as Kupffer cells, hepatic stellate cells (HSCs), and liver sinusoidal endothelial cells.^[^ ^86^, ^87^, ^88^ ^]^ In the future, we could explore adding further complexity by introducing other cell types, which may extend utility of the model to other types of studies. For example, fibrosis during NAFLD is a complex process that includes inflammation and multiple cell types, most notably HSCs that are known to be the main producers of fibrotic matrix.^[^ ^87^, ^89^ ^]^ This is likely why we observed no extracellular deposition of collagen within our cultures, despite up‐regulation of *COL1*. By integrating these cell types, we could develop a more comprehensive and physiologically relevant model of NAFLD that can better capture the multicellular interactions and disease progression. This will enable us to investigate the roles of these cell types in stiffness‐mediated lipid accumulation, fibrosis, and inflammation, as well as provide a more robust platform for drug screening and therapeutic development. Similarly, our current system does not include any vasculature structures, and instead relies on passive diffusion to provide sufficient oxygen and nutrients for organoids. More complex organoid models with coculture systems and/or vascular‐like structures would be particularly useful in studying how matrix stiffness influences crosstalk between different cell phenotypes.^[^ ^32^, ^90^, ^91^ ^]^ CARS provides a noninvasive method to visualize lipid droplets and their spatial positions, although it provides only qualitative information about their chemical composition. Our CARS observations suggest that acyl chain length may be impacted by matrix stiffness, which warrants further systematic investigation using metabolomics methods. In the future, coupling in vitro engineered model systems with metabolomics offers an exciting opportunity to explore the metabolic pathways associated with NAFLD.

A key technological advance that enabled the development of our new HO culture platform, was the use of a small molecule competitor to create a dynamic stiffening biomaterial. Without this technology, HOs were unable to reproducibly form in high stiffness matrices. The use of competitors not only decreased the initial stiffness for organoid formation, but also increased the gelation time for homogeneous mixing. In future work, the kinetics of the competitor can be tuned to achieve different stiffness recovery profiles, which could be computationally predicted through coupled reaction‐diffusion modeling.^[^ ^60^ ^]^ As changes in matrix stiffness is common across a wide range of different diseases,^[^ ^14^, ^92^, ^93^, ^94^ ^]^ this technological approach can be readily applied to other hydrogel systems with dynamic crosslinking to mimic the spatial and temporal changes that occur within the matrix during disease progression and aging.

## 4 Experimental Section

### HA Synthesis and Modification

Hyaluronic acid (HA) was functionalized using established methods.^[^ ^49^, ^60^, ^95^ ^]^ 1 wt% HA (60 kDa, LifeCore Biomedical HA 60 K) was dissolved in 2‐(*N*‐Morpholino) ethanesulfonic acid (MES) buffer (0.2 m MES hydrate (Sigma M2933) and 0.15 m NaCl in Milli‐Q water at pH 4.5). Propargylamine (Sigma P50900) was added to the reaction (6 eq. per HA unit group for 30% modification), and the pH was adjusted to 6 using 1N NaOH. *N*‐Hydroxysuccinimide (6 eq. to HA unit group; Thermo Fisher 24500) and EDC (6 eq. to HA unit group for 30% modification; Thermo Fisher 22980) were added into the reaction sequentially. The reaction was allowed to stir continuously for 24 h at RT. The solution was then dialyzed against Milli‐Q water for 3 d and lyophilized.

Benzaldehyde was conjugated to HA‐alkyne through a copper click reaction with azido‐benzaldehyde.^[^ ^49^, ^60^, ^95^ ^]^ Briefly, 1 wt% HA‐alkyne was dissolved in isotonic 10x phosphate‐buffered saline (10x PBS; 81 × 10^−3^ m sodium phosphate dibasic, 19 × 10^−3^ m sodium phosphate monobasic, 60 × 10^−3^ m sodium chloride in Milli‐Q water, pH 7.4) with 1 mg mL^−1^ beta‐cyclodextrin (Sigma C4767). The solution was degassed under nitrogen for 30 min after fully dissolved. 4.52 × 10^−3^ m sodium ascorbate (0.18 eq. per HA unit group, Sigma A7631) and 0.24 × 10^−3^ m copper (II) sulfate pentahydrate (0.0096 eq. per HA unit group, Sigma 209198) were prepared in Milli‐Q water and degassed for 30 min, and then the solutions were added sequentially to the HA‐alkyne mixture. Finally, azidobenzaldehyde (2.0 eq. to alkyne groups for ≈30% modification) in anhydrous DMSO (Sigma 276855) was introduced to HA‐alkyne solution and degassed for an additional 10 min. The reaction proceeded for 24 h under continuous stirring. An equal volume of 50 × 10^−3^ m ethylenediaminetetraacetic acid disodium salt dihydrate (EDTA, Fisher O2793–500) was added to chelate the remaining copper and terminate the reaction. The solution was then dialyzed, sterile‐filtered, lyophilized, and stored at −20 °C. The HA‐benzaldehyde was dissolved in D_2_O for nuclear magnetic resonance spectroscopy analysis (^1^H NMR, 500 MHz; Figure S1, Supporting Information). The modification percentage was determined by calculating the proton peaks of benzene ring (δ ppm 7.93 and 7.82, 2H each), triazole linkage (δ ppm 7.9, 1H) and aldehyde group (δ ppm 9.9, 1H) relative to the reference proton peaks of HA acetyl group (δ ppm 1.8, 3H).

### ELP Expression and Modification

Recombinant elastin‐like protein (ELP) was produced and purified as previously described.^[^ ^49^, ^60^, ^96^ ^]^ In summary, BL21(DE3) pLysS Escherichia coli (Invitrogen C606003) were transformed with pET15b plasmids carrying the ELP sequence with an RGD motif (Figure S2A, Supporting Information). The bacterial culture was grown in Terrific Broth (Thermo Fisher H26824.36) at 37 °C until reaching an optical density of 0.8 at 600 nm, at which point ELP expression was initiated with 1 × 10^−3^ m isopropyl β‐D‐1‐thiogalactopyranoside (Thermo Fisher BP1755). Following a 7‐h expression period at 32 °C, cells were harvested by centrifugation and lysed in TEN buffer (10 × 10^−3^ m Tris (Fisher BP152‐1), 1 × 10^−3^ m EDTA (Fisher BP2482100), and 100 × 10^−3^ m NaCl (Fisher BP358‐212), pH 8.0), supplemented with 10 × 10^−6^ m DNase I (Sigma DN25) and 1 × 10^−3^ m phenylmethanesulfonyl fluoride (PMSF; MP Biomedicals 195381) as a protease inhibitor. ELP was extracted from the bacterial pellet through three freeze–thaw cycles with alternating centrifugation steps at 4 and 37 °C. The final product underwent dialysis against Milli‐Q water for 3 d at 4 °C, was subsequently lyophilized, and stored at −20 °C.

The ELP hydrazine modification was following previously reported protocols.^[^ ^49^, ^60^, ^96^ ^]^ The lyophilized ELP was dissolved at 3 wt% in a 1:1 mixture of anhydrous dimethyl sulfoxide (DMSO; Sigma 276855) and anhydrous dimethylformamide (DMF; Sigma 227056). Tri‐Boc hydrazinoacetic acid (Sigma 68972) was dissolved in DMF at 2.1% w/v at 2 molar eq. per ELP amine groups. Hexafluorophosphate azabenzotriazole tetramethyl uronium (HATU; 2 eq. per ELP amine groups; Sigma 445460) and 4‐methylmorpholine (5 eq. per ELP amine groups; Sigma M56557) were added into the tri‐Boc hydrazinoacetic acid solution to activate for 10 min. Then the activated solution was slowly added to the ELP solution, and the reaction was allowed to proceed for 24 h at room temperature. The modified ELP was precipitated by ice‐cold diethyl ether (Fisher E138), collected via centrifugation (18000 g, 25 min) and dried overnight under nitrogen. The removal of Boc‐protecting groups was conducted by dissolving the modified ELP in a 1:1 mixture of dichloromethane (DCM; Sigma DX0835‐3) and trifluoroacetic acid (TFA; Sigma T6508) containing 5% v/v tri‐isopropylsilane (Sigma 233781) for 4 h at room temperature. The ELP‐hydrazine was precipitated, centrifuged, dried under nitrogen gas. The resulting samples were resuspended in water, dialyzed, sterile‐filtered and lyophilized, and was stored at −20 °C. The ELP products were dissolved in DMSO‐d6 for nuclear magnetic resonance spectroscopy analysis (1H NMR, 500 MHz; Figure S2B,C, Supporting Information). The modification percentage was determined by calculating the proton peaks of boc‐protected ELP and modified ELP tyrosine residues (δ ppm 7.00 and 6.62, 2H each); Boc groups on boc‐protected ELP (δ ppm 1.46 and 1.39, 27H groups).

### Hydrogel Rheological Characterization

Mechanical characterization of HELP hydrogels was performed using a stress‐controlled AR‐G2 rheometer (TA Instruments) with a 20 mm cone‐plate geometry (1° cone angle, 28 µm gap). All tests were conducted on 50 µL hydrogel samples, representative values are composed of 3–4 replicates per gel formulation.

4 wt% HA and 10 wt% ELP were dissolved in 10x PBS at 4 °C overnight before use. When forming a gel, HA and ELP solution were diluted to desired concentration, then 25 µL of the HA solution was pipetted onto the rheometer stage, then 25 µL of the ELP solution was pipetted directly into the droplet of HA and was quickly mixed. The geometry was immediately lowered to the geometry gap, and silicone oil was added to the gap to prevent dehydration. Hydrogel samples first reacted at 4 °C for 5 min under 1% oscillatory strain and 1 rad s^−1^ angular frequency, followed by a temperature ramp to 23 °C within 10 s, and another time sweep measurement at 23 °C for 15 min is performed. Finally, a last temperature ramp to 37 °C within 10 s was performed, and a final time sweep measurement at 37 °C for 15 min was completed under 1% oscillatory strain and 1 rad s^−1^ angular frequency. A frequency sweep from 0.1 to 100 Hz at 1% strain at 37 °C was performed to measure the elastic modulus of hydrogels. The reported shear modulus was the values from frequency sweep at 1 rad s^−1^ angular frequency.

### Gelation Time

25 µL HA and 25 µL ELP were quickly mixed using a pipette tip on the rheometer stage at 4 °C, then the geometry was immediately lowered to the geometry gap to start the modulus measurement. A timer was started upon mixing and the gelation time was indicated by the crossover point, where the storage modulus (*G*′) was observed to be equal or greater than the loss modulus (*G*′′). Representative values are composed of 3–4 replicates per condition.

### Rheological Characterization with Competitors

Butyraldehyde was prepared into 100 × 10^−3^ m stock solution in 10x PBS. Butyraldehyde was premixed with ELP solution for 30 min, and then mixed with HA solution. The final competitor concentration in HELP hydrogel was 10 × 10^−3^ m. Rheological characterization protocol was the same as described above. For the initial state of the HELP HI+ gel, HELP HI with 10 × 10^−3^ m butyraldehyde was directly mixed on the rheometer platform, and the storage modulus was measured at 37 °C, frequency sweep from 0.1 to 100 Hz and 1% strain. For the final state of the HELP HI+ gel, HELP HI with 10 × 10^−3^ m butyraldehyde was prepared in 8‐mm molds, then immersed in 1 mL PBS to let the competitor diffuse out of the gel and into the surrounding saline. The entire sample was kept at 37 °C, and surrounding PBS was refreshed every 3 d. On day 16, gels were scooped out from the molds, and stiffness was measured using oscillatory rheometer at 37 °C, frequency sweep from 0.1 to 100 Hz and 1% strain. The reported shear modulus was the values from frequency sweep at 1 rad s^−1^ angular frequency.

### Rheology Recovery During Competitor Release

HELP HI formulation 50 µL hydrogels were prepared with or without competitors on ice in 8 mm molds in a 24‐well plate. Hydrogels were kept on ice for 10 min, followed with 15 min at room temperature and 15 min at 37 °C. Then the samples were added with 1 mL of DPBS and incubated at 37 °C. DPBS was changed every 3 d.

Mechanical recovery characterization was performed on HELP hydrogels using a stress‐controlled AR‐G2 rheometer (TA Instruments) and an 8 mm plate geometry. The gels were gently moved from the molds using a spatula and moved to rheometer stage. The geometry head was lowered onto the sample until ≈1000 µm gap was achieved. The modulus of hydrogels were measured under a frequency sweep from 0.1 to 100 Hz at 1% strain at 37 °C. The final shear modulus was reported from the frequency sweep at 1 rad s^−1^ angular frequency. Representative values are replicates of 3–4 gels condition, and data was normalized by modulus of HELP HI without competitors on each day.

### Hepatic Spheroid Generation and Passaging in Matrigel

HOs were derived from iPSCs using previously reported methods.^[^ ^44^ ^]^ For all experiments, single cells are cultured into hepatoblast progenitor spheroids in growth media, then dissociated into single cells for passaging. Cells were used between passage of 5 and 15. Single iPSCs derived hepatoblast progenitor cells were cultured in 50 µL Matrigel (Corning, 356231) domes at 200 µL^−1^ seeding density within 24‐well plate to generate hepatic organoids. Spheroids were passaged every week. To passage spheroids, Matrigel domes were covered with 1 mL ice‐cold, 5 × 10^−3^ m EDTA for 30 min to disassociate the gel. Then the cell pellet was collected by centrifuging at 500× g for 5 min, and treated with TrypLE (Thermo Fisher Scientific) for 6 min at 37 °C. The TrypLE was then quenched with 40% FBS in PBS and centrifuged for 5 min at 500× g to collect the cell pellet. The cells were resuspended in growth medium, and the desired number of cells were collected and resuspended in Matrigel solution at 200 cells µL^−1^ on ice. The matrigel domes were allowed for gelation at 37 °C in the incubator for 10 min, then 700 µL of organoid growth medium was added to each well. 10 × 10^−6^ m Y‐27632 (Cayman Chemical 10005583) were added to the medium for the first media change. Media was changed every 2–3 d.

To make complete hepatic growth media, RPMI media was supplemented with 1x B27 (Thermo 17504044), 250 × 10^−9^ m LDN‐193189 (Cayman Chemical 19396), 3 × 10^−6^ m CHIR99021 (Cayman Chemical 13122), 10 × 10^−6^ m A83‐01 (Cayman Chemical 9001799), 100 ng mL^−1^ EGF (Cayman Chemical 32057), 10 ng mL^−1^ FGF10 (Cayman Chemical 32056), 20 ng mL^−l^ HGF (Cayman Chemical 32052).

### Hepatic Organoid Culture in HELP

Lyophilized HA and ELP were dissolved in 10x PBS at stock concentration of 5 and 12 wt%, respectively. For organoids cultured in HELP with competitors, butyraldehyde was pre‐mixed with ELP solution overnight before use. Hepatic spheroids were released from Matrigel and dissociated into single cell suspension as described above. 6 mg mL^−1^ laminin‐111 (R&D Systems 344600501) were added into ELP solution to achieve a final concentration of 0.1 wt% in all HELP formulations. The desired number of cells resuspended in stock solution of ELP and laminin. To form HELP gels, HA solution was first added to a custom 4 mm diameter silicone mold affixed to a glass cover slip within a 24 well plate as previously reported. Then calculated volume of ELP solution was pipetted into the molds and immediately mixed with HA. The hydrogels were incubated on ice for 10 min, followed by another 10 min at RT and a final 10 min at 37 °C. After complete gelation, 1 mL prewarmed growth media was added in each well and media change was performed every 2–3 d.

To make complete hepatic differentiation media, hepatic media (Lonza, CC 3198) was supplemented with 10 × 10^−6^ m DAPT (Cayman Chemical13197), 10 ng mL^−1^ oncostatin M (Peprotech 300–10), 20 ng mL^−1^ HGF (Cayman Chemical 32052), 10 × 10^−6^ m dexamethasone (Cayman Chemical 11015), 10 ng mL^−1^ BMP4 (Peprotech 120‐05ET) and 0.5% Penicillin–Streptomycin (Thermo Fisher Scientific 15140122). Differentiation media was added to spheroids on day 9 and was changed every 2–3 d for a week.

### Organoid Formation Efficiency and Diameter Quantification

Organoid formation efficiency and diameter were quantified using previously reported method.^[^ ^60^, ^94^ ^]^ To analyze organoid formation efficiency, brightfield images were taken under 20x magnification on day 3 via phase contrast using a THUNDER microscope (Leica Microsystems). For each gel, ≈150 µm z‐volume and 10 z‐slices were taken, and at least 3 non‐overlapping fields of view were chosen for analysis. Assuming uniform cell distribution, organoid formation efficiency was calculated by extrapolating the organoid numbers in the z‐stack volume in each view and comparing it to the initial cell seeding density. Averaged formation efficiency from 3 replicate cultures was reported. To measure organoid growth rate, hepatic organoids were imaged every 3 d from Day 0 until Day 16 at 10x magnification. 3 replicates and at least 9 non‐overlapping images were taken for each matrix stiffness condition. Organoid area was measured by manually drawing a circle over each organoid using ImageJ (NIH, v.2.1.0/1.53c), and diameter was calculated by assuming the shape of organoid a perfect circle.

### AlamarBlue Assay

Cell metabolic activity was measured using alamarBlue assay (Thermo Fisher Scientific A50100) to quantify the cell proliferation in different stiffness HELP matrices. Hepatic organoids were cultured in different HELP matrices, as outlined above. Following the assay instructions, alamarBlue was added into cell culture media and incubated at 37 °C for 4 h. Then 100 µL of media was taken out to measure the fluorescence at 560/590 nm (excitation/emission) using the plate reader. The data was normalized to the fluorescent signal on day 0. *N* = 3 replicate cultures for each condition were analyzed. Fresh media were changed to the cells for continuous cell culture.

### HELP Hydrogels Sectioning

Hepatic organoids were cultured in HELP gels in 5 mm molds as described above. Samples were fixed using 4% PFA for 15 min and three 10 min washes of PBS were performed. 30% sucrose solution was added to each sample and incubate at 4 °C overnight. Gels were placed into Tissue‐Tek Cryomold molds (Sakura Finetek USA, Torrance, CA), and then embedded into a 1 mL mixture of 30% sucrose and Tissue‐Tek O.C.T Compound (Sakura Finetek USA, Torrance, CA) at 1:1 ratio. After for 24 h incubation at RT, samples were moved onto dry ice for rapid freezing (≈10 min). The samples were then snap frozen on dry ice and cryo‐sectioned into 40 µm sections using a Leica Cryostat instrument.

Sectioned samples were melted at 50 °C for 5–10 min and subsequently washed with DPBS to melt and removed excess O.C.T. The gel sections were circled with lipid‐repellent marker pen for further staining. The staining process was described as below. The stained samples were mounted to No.1 glass over slides with ProLong Gold Antifade Reagent for 24–48 h at RT in dark. Stained samples were imaged using a confocal microscope (Leica SPE) and image analysis was done by ImageJ (NIH, v.2.1.0/1.53c)

### Histology and Immunocytochemistry

Organoids in HELP gels were collected and fixed for 15 min at room temperature in 4% paraformaldehyde (PFA). The samples were then embedded in HistoGel (ThermoFisher, USA) and followed by paraffin. The samples were cut into 8 µm of sections and deparaffinized. Hematoxylin and eosin (H&E) and Sirius Red staining was performed according to standard protocols.

Organoids were fixed with 750 µL of 4% PFA for 15 min and washed for three times 10 min by DPBS. Cells were permeabilized with 0.5% Triton X‐100 for 1 h, then blocked for 3 h in blocking solution (5% goat serum, 0.5% Triton X‐100 and 5 wt% BSA in DPBS). Primary antibodies (**Table** **1**) were prediluted in DPBS with antibody dilution solution (2.5% goat serum, 0.5% Triton X‐100 and 2.5 wt% BSA) at 4 °C overnight. Samples were washed with PBST (DPBS with 0.1% Triton X‐100) three times for 20 min before secondary antibodies were added. Secondary antibodies were diluted at 1:500 in antibody dilution solution and samples were incubated at 4 °C overnight. After three times 20 min PBST wash, samples were stained with DAPI (5 mg mL^−1^) at 1:2000 dilution and tetramethylrhodamine (TRITC)‐phalloidin (100 µg mL^−1^) at 1:400 dilution in PBST overnight at 4 °C. Samples then were washed with PBST (3×5 min) and mounted to No.1 glass over slides with ProLong Gold Antifade Reagent for 24–48 h at RT. Stained samples were imaged using a confocal microscope (Leica SPE) and image analysis was done by ImageJ (NIH, v.2.1.0/1.53c).

**Table 1:** Antibody information.

Target	Species	Vendor	Product #	Dilution
Ki67	Rabbit	Cell Signal. Tech	12202	1:200
Cytokeratin 19	Mouse	DAKO	M088801‐2	1:50
HNF4A	Rabbit	Invitrogen	PA5‐82159	1:100
CYP3A4	Mouse	Santa Cruz Biotechnology	Sc‐53850	1:50
COL‐1	Mouse	Santa Cruz Biotechnology	sc‐59772	1:100
PLIN2	Rabbit	Abcam	Ab52356	1:100
E‐Cadherin	Rabbit	Cell Signal. Tech	3159	1:400

### Albumin Production

Albumin production was measured using human albumin ELISA kit (Abcam 108788). Hepatic organoids were cultured in different HELP matrices as outlined above. After 16 d of culture, cell supernatant was collected for albumin production analysis by following the assay instructions. *N* = 4 replicate cultures for each condition were analyzed. Organoids were released from HELP gels and disassociated into single cells for cell counting. The final albumin production was normalized to cell numbers in each gel.

### Urea Production

Urea production was measured using human urea assay kit (Abcam 83362). Hepatic organoids were cultured in different HELP matrices as outlined above. After 16 d of culture, cell supernatant was collected for urea production analysis by following the assay instructions. *N* = 3 replicate cultures for each condition were analyzed. Organoids were released from HELP gels and disassociated into single cells for cell counting. The final albumin production was normalized to cell numbers in each gel.

### Rhodamine 123 Transportation Assay

Hepatic organoids were cultured in different stiffness HELP for 16 d of maturation. To test the activity of multidrug resistant protein 1 (MRP1) transporter, organoids were treated with 100 × 10^−6^ m rhodamine 123 (Thermo Fisher) for 30 min in differentiation media at 37 °C. The organoids were then washed with DPBS for three times. For control groups, organoids were treated with 20 × 10^−6^ m verapamil (Sigma‐Aldrich) for 30 min before addition of rhodamine 123 to inhibit MRP1‐dependent transporter activity. The fluorescent organoids were imaged with a Leica Thunder microscope (Leica Microsystems, THUNDER Imager 3D Cell Culture) at excitation 488 nm and emission 529 nm.

### Quantitative Reverse Transcription Polymerase Chain Reaction (qRT‐PCR)

Targeted quantification of mRNA expression was achieved with qRT‐PCR as reported before.^[^ ^95^ ^]^ First, hydrogels were treated with equal volume of elastase (500 U mL^−1^; GoldBio) and hyaluronidase (2000 U mL^−1^; Sigma‐Aldrich) for 1 h at 37 °C to release encapsulated organoids from HELP. The samples were then resuspended in 500 µL of TRIzol reagent (Invitrogen) and stored in −80 °C freezer until use. For RNA extraction, the samples were disrupted for 3 times with probe sonication (Heilscher UP50H, 50% amplitude, 30‐kHz frequency, 0.5‐s cycle). mRNA was extracted by phenol‐chloroform and 5PRIME phase lock gels (Quantabio) and purified by isopropyl alcohol precipitation and ethanol washes. The samples were dried at RT for 1 h and dissolved in nuclease free water. The mRNA concentration was measured using NanoDrop (Thermo Fisher Scientific), and 50–100 ng of mRNA was reverse transcribed using the High‐Capacity cDNA Reverse Transcription Kit (Applied Biosystems). 5 × 10^−6^ m stock solution of primer pairs (Integrated DNA Technologies; Table S1, Supporting Information) was prepared in advance and stored in −20 °C. qPCR was performed on 6.6 µL cDNA mixed with 0.9 µL solution of primer pairs and 7.5 µL of Fast SYBR Green Master Mix (Applied Biosystems). Reactions was run with the StepOnePlus Real Time PCR System (Applied Biosystems). Cycle threshold (CT) values were calculated using the StepOnePlus software (v.2.3) and normalized to analyzed by β‐actin using the ΔCT method.

### Oleic Acid Treatment

Oleic acid (in ethanol, Cayman Chemicals 90260,) was added at desired dilution (5 mL differentiation media + 1.41 µL oleic acid) to prewarmed hepatic organoid differentiation media and stirred for 1–3 h at 37 °C. Control media was prepared by adding the same volume of ethanol. Hepatic organoids cultured in HELP were added 1 mL differentiation media with or without oleic acid and cultured for 3 d in the incubator.

### BODIPY Staining of Lipid Droplets

BODIPY 493/503 (ThermoFisher, D3922) was dissolved in DMSO at 5 × 10^−3^ m stock solution until use. For staining, BODIPY stock solution was diluted at 1:2500 in PBS every time before use. Samples were washed with DPBS for 5 min, three times, and BODIPY staining solution was added and incubated at 37 °C for 30 min. Three times DPBS washing was performed and then the samples were fixed using 4% PFA for 15 min. Fixation solution was then removed and three 10 min washes of DPBS were performed. Samples were stained with DAPI (5 mg mL^−1^) at 1:2000 dilution and tetramethylrhodamine (TRITC)‐phalloidin (100 µg mL^−1^) at 1:400 dilution in PBST overnight at 4 °C. Samples then were washed with PBST (3×10 min) and imaged using a confocal microscope (Leica SPE) and analyzed using ImageJ (NIH, v.2.1.0/1.53c). Lipid percentage quantification was based on 2D area of max projection. Lipid droplets were quantified using BODIPY staining lipid area, and organoid area was quantified by phalloidin staining organoid area.

### Triglyceride Assay

Triglyceride quantification in organoids was measured using Triglyceride‐Glo assay (Promega J3160). Hydrogels were treated with equal volume of elastase (500 U mL^−1^; GoldBio) and hyaluronidase (2000 U mL^−1^; Sigma‐Aldrich) for 1 h at 37 °C to release encapsulated organoids from HELP. Organoids were disassociated into single cells with TrypLE and centrifuged for cell counting. The resulted cells were lysed, and triglyceride concentration was measured for different conditions by following the assay instructions. The final triglyceride concentrations were normalized to cell numbers in each gel and averaged through *N* = 4 replicates for each condition.

### CARS Setup and Data Collection

Lipid droplets were mapped in the HOs by probing CARS signals of their CH stretching vibrations using an inverted microscope (Nikon, Ti2‐E equipped with a C2 confocal scanning head and a Nikon CFI Plan Apo IR 60XC water immersion objective, NA = 1.27). The scanner was equipped with a dual‐beam input module (Optique Peter) that allowed switching between linear and nonlinear excitation modes for overlapping scanning confocal fluorescence images. In CARS acquisitions, CH vibrations were coherently excited by the interaction of two temporally and spatially overlapping laser beams. These beams were produced by a picosecond‐pulsed laser system comprising a 1031 nm fiber laser and an optical parametric oscillator (OPO) tunable between 690–960 nm (APE picoEmerald S, 2 ps pulse duration, 80 MHz repetition rate, and 10 cm⁻¹ bandwidth). The OPO wavelength was set to 797 nm to address the methylene symmetric stretching vibrations at 2850 cm^−1^. The methyl stretching vibrations at 2930 cm^−1^ was measured by tuning the OPO to 791.8 nm. 55 mW excitation powers of the OPO beam and 32 mW of the 1031 nm Stokes beam were used for the samples. The CARS signals were collected in the forward direction using a photomultiplier tube (Hamamatsu, R6357) equipped with hard‐coated optical filters (Semrock, one shortpass FF01‐750/SP and two bandpass FF01‐643/20). The dwell time was ≈5.5 µs per pixel and the pixel size was <200 nm. The 1031 nm Stokes beam at 20 MHz was amplitude‐modulated and CARS signal was isolated with a lock‐in‐amplifier (Zürich Instruments, HF2LI) synced at this modulation frequency to minimize the impact of background signals. For each organoid, stacks with >15 steps and 0.5 µm step size were acquired. For measurements of full spectra in the CH stretching region, sequential stacks were collected for which the OPO wavelength was tuned between 801 and 785.5 nm with a step size of 0.5 nm (32 steps in total). The excitation powers were reduced by ≈30% for the full spectral acquisitions to minimize the risk of photodamage.

The acquired images were prepared and analyzed in ImageJ. The lipid droplet maps were generated by treating the images captured at 2850 cm^−1^ with a gaussian blur filter (sigma = 1.2) and background subtraction with a rolling ball radius of 30 pixels, followed by thresholding initially with the Intermodes routine and manual optimization to ensure all lipid droplets were included. The lipid droplets were separated with the Disconnect Particles plugin and the resulting mask was used in subsequent analyses, including size evaluation by extracting the lipid droplet volumes with the 3D Objects Counter plugin. The average signal within the boundaries for each isolated feature in the mask was determined at each wavenumber using the Intensity Measurements 2D/3D plugin. Acyl chain length maps was produced by forming maps where each droplet is assigned its average intensities at 2850 cm^−1^ and 2930 cm^−1^ and then taking the ratio between them. The script for image analysis was supplemented in Methods S1 in the Supporting Information.

### Lactate Dehydrogenase‐Metric of Cytotoxicity

The cytotoxicity of Y27632 was performed using LDH‐GloTM Cytotoxicity Assay (Promega J2380). HOs were cultured in HELP formulations for 16 d as outlined above. On day 16, organoids were treated either with 20 or 0 × 10^−6^ m Y27632 for 3 d. LDH storage buffer (200 × 10^−3^ m Tris‐HCl (pH 7.3), 10% Glycerol, 1% BSA) was prepared according to the assay instructions. 10 µL of culture media was stored in LDH storage buffer at −80 °C until measurement. Percentage cytotoxicity was normalized to HELP formulation with no Y27632. *N* = 4 replicates for each condition were analyzed.

### Statistical Analysis

The statistical significance was used for all tests: * = *p* < 0.05, ** = *p* < 0.01, *** = *p* < 0.001, **** = *p* < 0.0001. All the data presented are mean with standard deviation. Data from Figures 1, 2, 3, 4, 6, and 7b were analyzed by one‐way analysis of variance (ANOVA) with Tukey's multiple comparisons testing to compare individual means. The data from Figures 3, 5, and 7c were analyzed via unpaired, two‐tailed Student's *t*‐test. The data from Figures 4e and 6c,g were analyzed by two‐way ANOVA with Tukey's multiple comparisons testing to compare individual means. The data in Figures S3,S9, and S13 in the Supporting Information were analyzed using one‐way ANOVA with Tukey's multiple comparisons testing. The data in Figures S10,S11, and S12 (Supporting Information) were analyzed via unpaired, two‐tailed Student's *t*‐test. All statistical analysis was performed using GraphPad Prism 10.0 software (GraphPad Software).

## Conflict of Interest

The authors declare no conflict of interest.

## Supporting information



Supporting Information

## Data Availability

The data that support the findings of this study are available from the corresponding author upon reasonable request.

## References

[1] You , Z. M. nossi , A. B. Koenig , D. Abdelatif , Y. Fazel , L. Henry , M. Wymer , Hepatology 2016, 64, 73. 26707365 10.1002/hep.28431

[2] H. Jarvis , D. Craig , R. Barker , G. Spiers , D. Stow , Q. M. Anstee , B. Hanratty , PLoS Med. 2020, 17, 1003100. 10.1371/journal.pmed.1003100 PMC7192386 32353039

[3] V. W. Wong , A. K. Singal , Transl. Gastroenterol. Hepatol. 2019, 4, 3.30854490

[4] K. Riazi , H. Azhari , J. H. Charette , F. E. Underwood , J. A. King , E. E. Afshar , M. G. Swain , S. E. Congly , G. G. Kaplan , A.‐A. Shaheen , Lancet Gastroenterol. Hepatol. 2022, 7, 851. 35798021 10.1016/S2468-1253(22)00165-0

[5] E. Guirguis , J. Dougherty , K. Thornby , Y. Grace , K. Mack , Ann. Pharmacother. 2025, 59, 162. 38887011 10.1177/10600280241259528

[6] S. A. Harrison , P. Bedossa , C. D. Guy , J. M. Schattenberg , R. Loomba , R. Taub , D. Labriola , S. E. Moussa , G. W. Neff , M. E. Rinella , Q. M. Anstee , M. F. Abdelmalek , Z. Younossi , S. J. Baum , S. Francque , M. R. Charlton , P. N. Newsome , N. Lanthier , I. Schiefke , A. Mangia , J. M. Pericàs , R. Patil , A. J. Sanyal , M. Noureddin , M. B. Bansal , N. Alkhouri , L. Castera , M. Rudraraju , V. Ratziu , N. Engl. J. Med. 2024, 390, 497. 38324483 10.1056/NEJMoa2309000

[7] P. S. Dulai , S. Singh , J. Patel , M. Soni , L. J. Prokop , Z. Younossi , G. Sebastiani , M. Ekstedt , H. Hagstrom , P. Nasr , P. Stal , V. W. S. Wong , S. Kechagias , R. Hultcrantz , R. Loomba , Hepatology 2017, 65, 1557. 28130788 10.1002/hep.29085 PMC5397356

[8] F. Marra , A. Gastaldelli , G. Svegliati Baroni , G. Tell , C. Tiribelli , Trends Mol. Med. 2008, 14, 72. 18218340 10.1016/j.molmed.2007.12.003

[9] R. Loomba , S. L. Friedman , Cell 2021, 184, 2537. 33989548 10.1016/j.cell.2021.04.015 PMC12168897

[10] S. Friedman , M. Pinzani , Hepatology 2021, 75, 73. 10.1002/hep.32285 PMC12179971 34923653

[11] J. Herrera , C. A. Henke , P. B. Bitterman , J. Clin. Invest. 2018, 128, 45. 29293088 10.1172/JCI93557 PMC5749528

[12] S. L. Friedman , B. A. Neuschwander‐Tetri , M. Rinella , A. J. Sanyal , Nat. Med. 2018, 24, 908. 29967350 10.1038/s41591-018-0104-9 PMC6553468

[13] Y. Geng , K. N. Faber , V. E. de Meijer , H. Blokzijl , H. Moshage , Hepatol. Int. 2021, 15, 21. 33548031 10.1007/s12072-020-10121-2 PMC7886759

[14] C. Bonnans , J. Chou , Z. Werb , Nat. Rev. Mol. Cell Biol. 2014, 15, 786. 25415508 10.1038/nrm3904 PMC4316204

[15] S. R. Caliari , M. Perepelyuk , B. D. Cosgrove , S. J. Tsai , G. Y. Lee , R. L. Mauck , R. G. Wells , J. A. Burdick , Sci. Rep. 2016, 6, 21387. 26906177 10.1038/srep21387 PMC4764908

[16] T. Xia , R. Zhao , F. Feng , L. Yang , Polymers 2020, 12, 1903. 32846973 10.3390/polym12091903 PMC7564768

[17] L. Chin , N. D. Theise , A. E. Loneker , P. A. Janmey , R. G. Wells , Am. J. Physiol.‐Gastroint. Liver Physiol. 2020, 319, G11. 10.1152/ajpgi.00098.2020 PMC7468756 32463334

[18] F. Baldini , M. Khalil , A. Bartolozzi , M. Vassalli , A. Di Ciaula , P. Portincasa , L. Vergani , Biomolecules 2022, 12, 733. 35625660 10.3390/biom12050733 PMC9139073

[19] Y.‐S. Lee , S. E. In , Cell. Mol. Gastroenterol. Hepat. 2023, 16, 355. 10.1016/j.jcmgh.2023.05.010 PMC10444957 37270060

[20] A. M. Carvalho , R. Bansal , C. C. Barrias , B. Sarmento , Adv. Mater. 2024, 36, 2307673. 10.1002/adma.202307673 37961933

[21] C. J. Green , S. A. Parry , P. J. Gunn , C. D. L. Ceresa , F. Rosqvist , M.‐E. Piché , L. Hodson , Horm. Mol. Biol. Clin. Invest. 2020, 41. 10.1515/hmbci-2018-0038 30098284

[22] L. Hebbard , J. George , Nat. Rev. Gastroenterol. Hepatol. 2011, 8, 35. 21119613 10.1038/nrgastro.2010.191

[23] Y. R. Im , H. Hunter , G. de , D. Hahn , A. Duret , Q. Cheah , J. Dong , M. Fairey , C. Hjalmarsson , A. Li , H. K. Lim , L. McKeown , C. G. Mitrofan , R. Rao , M. Utukuri , I. A. Rowe , J. P. Mann , Hepatology 2021, 74, 1884. 33973269 10.1002/hep.31897

[24] Y. Takahashi , Y. Soejima , T. Fukusato , World J. Gastroenterol. 2012, 18, 2300. 22654421 10.3748/wjg.v18.i19.2300 PMC3353364

[25] M. Elbadawy , M. Yamanaka , Y. Goto , K. Hayashi , R. Tsunedomi , S. Hazama , H. Nagano , T. Yoshida , M. Shibutani , R. Ichikawa , J. Nakahara , T. Omatsu , T. Mizutani , Y. Katayama , Y. Shinohara , A. Abugomaa , M. Kaneda , H. Yamawaki , T. Usui , K. Sasaki , Biomaterials 2020, 237, 119823. 32044522 10.1016/j.biomaterials.2020.119823

[26] M. J. Ramos , L. Bandiera , F. Menolascina , J. A. Fallowfield , iScience 2022, 25, 103549. 34977507 10.1016/j.isci.2021.103549 PMC8689151

[27] Y. Liu , A. E. Gilchrist , S. C. Heilshorn , Adv. Mater. 2407794. 10.1002/adma.202407794 PMC11573243 39233559

[28] B. L. LeSavage , R. A. Suhar , N. Broguiere , M. P. Lutolf , S. C. Heilshorn , Nat. Mater. 2022, 21, 143. 34385685 10.1038/s41563-021-01057-5 PMC12276900

[29] M. W. Tibbitt , K. S. Anseth , Biotechnol. Bioeng. 2009, 103, 655. 19472329 10.1002/bit.22361 PMC2997742

[30] Y. Wang , H. Wang , P. Deng , T. Tao , H. Liu , S. Wu , W. Chen , J. Qin , ACS Biomater. Sci. Eng. 2020, 6, 5734. 33320545 10.1021/acsbiomaterials.0c00682

[31] J. Wu , P. Li , C. Dong , H. Jiang , X. Bin , X. Gao , M. Qin , W. Wang , C. Bin , Y. Cao , Nat. Commun. 2018, 9, 620. 29434258 10.1038/s41467-018-02917-6 PMC5809592

[32] R. Ouchi , S. Togo , M. Kimura , T. Shinozawa , M. Koido , H. Koike , W. Thompson , R. A. Karns , C. N. Mayhew , P. S. McGrath , H. A. McCauley , R.‐R. Zhang , K. Lewis , S. Hakozaki , A. Ferguson , N. Saiki , Y. Yoneyama , I. Takeuchi , Y. Mabuchi , C. Akazawa , H. Y. Yoshikawa , J. M. Wells , T. Takebe , Cell Metab. 2019, 30, 374. 31155493 10.1016/j.cmet.2019.05.007 PMC6687537

[33] D. Hendriks , J. F. Brouwers , K. Hamer , M. H. Geurts , L. Luciana , S. Massalini , C. López‐Iglesias , P. J. Peters , M. J. Rodríguez‐Colman , S. Chuva de Sousa Lopes , B. Artegiani , H. Clevers , Nat. Biotechnol. 2023, 41, 1567. 36823355 10.1038/s41587-023-01680-4 PMC10635827

[34] A. Ravichandran , B. Murekatete , D. Moedder , C. Meinert , L. J. Bray , Sci. Rep. 2021, 11, 15566. 34330947 10.1038/s41598-021-94990-z PMC8324893

[35] H. K. Kleinman , G. R. Martin , Semin. Cancer Biol. 2005, 15, 378. 15975825 10.1016/j.semcancer.2005.05.004

[36] J. Glowacki , S. Mizuno , Biopolymers 2008, 89, 338. 17941007 10.1002/bip.20871

[37] N. Gjorevski , N. Sachs , A. Manfrin , S. Giger , M. E. Bragina , P. Ordóñez‐Morán , H. Clevers , M. P. Lutolf , Nature 2016, 539, 560. 27851739 10.1038/nature20168

[38] D. R. Hunt , K. C. Klett , S. Mascharak , H. Wang , D. Gong , J. Lou , X. Li , P. C. Cai , R. A. Suhar , J. Y. Co , B. L. LeSavage , A. A. Foster , Y. Guan , M. R. Amieva , G. Peltz , Y. Xia , C. J. Kuo , Adv. Sci. 2021, 8, 2004705. 10.1002/advs.202004705 PMC8132048 34026461

[39] V. Hernandez‐Gordillo , T. Kassis , A. Lampejo , G. Choi , M. E. Gamboa , J. S. Gnecco , A. Brown , D. T. Breault , R. Carrier , L. G. Griffith , Biomaterials 2020, 254, 120125. 32502894 10.1016/j.biomaterials.2020.120125 PMC8005336

[40] F. M. Yavitt , B. E. Kirkpatrick , M. R. Blatchley , K. F. Speckl , E. Mohagheghian , R. Moldovan , N. Wang , P. J. Dempsey , K. S. Anseth , Sci. Adv. 2023, 9, add5668. 10.1126/sciadv.add5668 PMC9858500 36662859

[41] S. Ye , J. W. B. Boeter , M. Mihajlovic , F. G. van Steenbeek , M. E. van Wolferen , L. A. Oosterhoff , A. Marsee , M. Caiazzo , L. J. W. van der Laan , L. C. Penning , T. Vermonden , B. Spee , K. Schneeberger , Adv. Funct. Mater. 2020, 30, 2000893. 34658689 10.1002/adfm.202000893 PMC7611838

[42] G. Sorrentino , S. Rezakhani , E. Yildiz , S. Nuciforo , M. H. Heim , M. P. Lutolf , Nat. Commun. 2020, 11, 3416. 32651372 10.1038/s41467-020-17161-0 PMC7351772

[43] M. Krüger , L. A. Oosterhoff , M. E. van Wolferen , S. A. Schiele , A. Walther , N. Geijsen , L. De Laporte , L. J. W. van der Laan , L. M. Kock , Adv. Healthcare Mater. 2020, 9, 1901658. 10.1002/adhm.201901658 32090504

[44] Y. Guan , D. Xu , P. M. Garfin , U. Ehmer , M. Hurwitz , G. Enns , S. Michie , M. Wu , M. Zheng , T. Nishimura , J. Sage , G. Peltz , JCI Insight 2023, 2. 10.1172/jci.insight.94954 PMC5621886 28878125

[45] Y. Guan , A. Enejder , M. Wang , Z. Fang , L. Cui , S.‐Y. Chen , J. Wang , Y. Tan , M. Wu , X. Chen , P. K. Johansson , I. Osman , K. Kunimoto , P. Russo , S. C. Heilshorn , G. Peltz , Nat. Commun. 2021, 12, 6138. 34686668 10.1038/s41467-021-26410-9 PMC8536785

[46] Y. Guan , G. Peltz , Liver International 2024, 44, 1290. 38451053 10.1111/liv.15893

[47] Y. Guan , Z. Fang , A. Hu , S. Roberts , P. K. Johansson , S. C. Heilshorn , A. Enejder , G. Peltz , bioRxiv 2023, 2023.04.25.538102.

[48] H. Wang , D. Zhu , A. Paul , L. Cai , A. Enejder , F. Yang , S. C. Heilshorn , Adv. Funct. Mater. 2017, 27, 1605609. 33041740 10.1002/adfm.201605609 PMC7546546

[49] M. E. Hefferon , M. S. Huang , Y. Liu , R. S. Navarro , N. de Paiva Narciso , D. Zhang , G. Aviles‐Rodriguez , S. C. Heilshorn , Curr. Protoc. 2023, 3, 917. 10.1002/cpz1.917 PMC10629846 37929691

[50] R. A. Suhar , V. M. Doulames , Y. Liu , M. E. Hefferon , O. Figueroa , H. Buabbas , S. C. Heilshorn , Biomater. Sci. 2022, 10, 2590. 35411353 10.1039/d1bm01890f PMC9123900

[51] A.‐M. Mustonen , A. Salvén , V. Kärjä , K. Rilla , J. Matilainen , P. Nieminen , Glycobiology 2019, 29, 298. 30689936 10.1093/glycob/cwz002

[52] R. A. Suhar , M. S. Huang , R. S. Navarro , G. Aviles Rodriguez , S. C. Heilshorn , Biomacromolecules 2023, 24, 5926. 37988588 10.1021/acs.biomac.3c00941

[53] J. Kanta , Front. Physiol. 2016, 7.

[54] S. Roberts , M. Dzuricky , A. Chilkoti , FEBS Lett. 2015, 589, 2477. 26325592 10.1016/j.febslet.2015.08.029 PMC4599720

[55] B. Wang , S. S. Patkar , K. L. Kiick , Macromol. Biosci. 2021, 21, 2100129. 10.1002/mabi.202100129 PMC8449816 34145967

[56] M. Yin , J. Woollard , X. Wang , V. E. Torres , P. C. Harris , C. J. Ward , K. J. Glaser , A. Manduca , R. L. Ehman , Magn. Reson. Med. 2007, 58, 346. 17654577 10.1002/mrm.21286

[57] M. Yoneda , K. Fujita , M. Inamori , A. Nakajima , M. Tamano , H. Hiraishi , Gut 2007, 56, 1330. 17470477 10.1136/gut.2007.126417 PMC1954961

[58] S. VNd , M. M. B. Leite‐Mór , M. Kondo , J. R. Martins , H. Nader , V. P. Lanzoni , E. R. Parise , Braz. J. Med. Biol. Res. 2005, 38, 747. 15917956 10.1590/s0100-879x2005000500012

[59] P. Sorrentino , L. Terracciano , S. D'Angelo , U. Ferbo , A. Bracigliano , R. Vecchione , ACG 2010, 105, 336. 10.1038/ajg.2009.587 19861959

[60] A. E. Gilchrist , Y. Liu , K. Klett , Y.‐C. Liu , S. Ceva , S. C. Heilshorn , Chem. Mater. 2023, 35, 8969.

[61] K. Watanabe , M. Ueno , D. Kamiya , A. Nishiyama , M. Matsumura , T. Wataya , J. B. Takahashi , S. Nishikawa , S. Nishikawa , K. Muguruma , Y. Sasai , Nat. Biotechnol. 2007, 25, 681. 17529971 10.1038/nbt1310

[62] H. Sendi , I. Mead , M. Wan , M. Mehrab‐Mohseni , K. Koch , A. Atala , H. Bonkovsky , C. Bishop , PLoS One 2018, 13, 0200847. 10.1371/journal.pone.0200847 PMC6053181 30024933

[63] J. E. Chin , R. Soffir , K. E. Noonan , K. Choi , I. B. Roninson , Mol. Cell. Biol. 1989, 9, 3808. 2571078 10.1128/mcb.9.9.3808-3820.1989 PMC362442

[64] N. Stefan , H.‐U. Häring , K. Cusi , Lancet Diab. Endocrinol. 2019, 7, 313. 10.1016/S2213-8587(18)30154-2 30174213

[65] O. Quehenberger , A. M. Armando , A. H. Brown , S. B. Milne , D. S. Myers , A. H. Merrill , S. Bandyopadhyay , K. N. Jones , S. Kelly , R. L. Shaner , C. M. Sullards , E. Wang , R. C. Murphy , R. M. Barkley , T. J. Leiker , C. R. H. Raetz , Z. Guan , G. M. Laird , D. A. Six , D. W. Russell , J. G. McDonald , S. Subramaniam , E. Fahy , E. A. Dennis , J. Lipid Res. 2010, 51, 3299. 20671299 10.1194/jlr.M009449 PMC2952570

[66] G. Musso , R. Gambino , M. Cassader , Prog. Lipid Res. 2009, 48, 1. 18824034 10.1016/j.plipres.2008.08.001

[67] J.‐X. Cheng , X. S. Xie , J. Phys. Chem. B 2004, 108, 827.

[68] J. Kiskis , H. Fink , L. Nyberg , J. Thyr , J.‐Y. Li , A. Enejder , Sci. Rep. 2015, 5, 13489. 26311128 10.1038/srep13489 PMC4550829

[69] H. A. Rinia , K. N. J. Burger , M. Bonn , M. Müller , Biophys. J. 2008, 95, 4908. 18689461 10.1529/biophysj.108.137737 PMC2576358

[70] D. L. Gorden , D. S. Myers , P. T. Ivanova , E. Fahy , M. R. Maurya , S. Gupta , J. Min , N. J. Spann , J. G. McDonald , S. L. Kelly , J. Duan , M. C. Sullards , T. J. Leiker , R. M. Barkley , O. Quehenberger , A. M. Armando , S. B. Milne , T. P. Mathews , M. D. Armstrong , C. Li , W. V. Melvin , R. H. Clements , M. K. Washington , A. M. Mendonsa , J. L. Witztum , Z. Guan , C. K. Glass , R. C. Murphy , E. A. Dennis , A. H. Merrill , et al., J. Lipid Res. 2015, 56, 722. 25598080 10.1194/jlr.P056002 PMC4340319

[71] D. H. Ipsen , J. Lykkesfeldt , P. Tveden‐Nyborg , Cell. Mol. Life Sci. 2018, 75, 3313. 29936596 10.1007/s00018-018-2860-6 PMC6105174

[72] M. Kohjima , M. Enjoji , N. Higuchi , M. Kato , K. Kotoh , T. Yoshimoto , T. Fujino , M. Yada , R. Yada , N. Harada , R. Takayanagi , M. Nakamuta , Int. J. Mol. Med. 2007, 20, 351. 17671740

[73] S. BasuRay , Y. Wang , E. Smagris , J. C. Cohen , H. H. Hobbs , Proc. Natl. Acad. Sci. USA 2019, 116, 9521. 31019090 10.1073/pnas.1901974116 PMC6511016

[74] S. Violante , L. IJlst , B. Ht , I. T. de Almeida , R. J. A. Wanders , F. V. Ventura , S. M. Houten , FASEB J. 2013, 27, 2039. 23322164 10.1096/fj.12-216689

[75] P. Targett‐Adams , D. Chambers , S. Gledhill , R. G. Hope , J. F. Coy , A. Girod , J. McLauchlan , J. Biol. Chem. 2003, 278, 15998. 12591929 10.1074/jbc.M211289200

[76] S. M. Johnson , H. Bao , C. E. McMahon , Y. Chen , S. D. Burr , A. M. Anderson , K. Madeyski‐Bengtson , D. Lindén , X. Han , J. Liu , Nat. Commun. 2024, 15, 4847. 38844467 10.1038/s41467-024-49224-x PMC11156938

[77] Q. Wu , J. Liu , L. Liu , Y. Chen , J. Wang , L. Leng , Q. Yu , Z. Duan , Y. Wang , ACS Biomater. Sci. Eng. 2018, 4, 3016. 33435021 10.1021/acsbiomaterials.8b00652

[78] S. J. Woolsey , S. E. Mansell , R. B. Kim , R. G. Tirona , M. D. Beaton , Drug Metab. Dispos. 2015, 43, 1484. 26231377 10.1124/dmd.115.065979

[79] F. P. Guengerich , Annu. Rev. Pharmacol. Toxicol. 1999, 39, 1. 10331074 10.1146/annurev.pharmtox.39.1.1

[80] B. Nie , H. M. Park , M. Kazantzis , M. Lin , A. Henkin , S. Ng , S. Song , Y. Chen , H. Tran , R. Lai , C. Her , J. J. Maher , B. M. Forman , A. Stahl , Hepatology 2012, 56, 1300. 22531947 10.1002/hep.25797 PMC3445775

[81] P. Romani , L. Valcarcel‐Jimenez , C. Frezza , S. Dupont , Nat. Rev. Mol. Cell Biol. 2021, 22, 22. 33188273 10.1038/s41580-020-00306-w

[82] K. Burridge , E. Monaghan‐Benson , D. M. Graham , Philos. Trans. Royal Soc. B 2019, 374, 20180229. 10.1098/rstb.2018.0229 PMC6627015 31431179

[83] X. Chen , X.‐R. Tan , S.‐J. Li , X.‐X. Zhang , Life Sci. 2019, 235, 116829. 31484042 10.1016/j.lfs.2019.116829

[84] I. Sousa‐Lima , H. J. Kim , J. Jones , Y‐B. Kim , Rho‐ Diab. Metab. J. 2021, 45, 655. 10.4093/dmj.2021.0197 PMC8497927 34610720

[85] J. M. Ramos Pittol , A. Milona , I. Morris , E. C. L. Willemsen , S. W. van der Veen , E. Kalkhoven , S. W. C. van Mil , Gastroenterology 2020, 159, 1853. 32712104 10.1053/j.gastro.2020.07.036

[86] N. Duarte , I. C. Coelho , R. S. Patarrão , J. I. Almeida , C. Penha‐Gonçalves , M. P. Macedo , Biomed Res. Int. 2015, 2015, 984578. 26090470 10.1155/2015/984578 PMC4450298

[87] P. Trivedi , S. Wang , S. L. Friedman , Cell Metab. 2021, 33, 242. 33232666 10.1016/j.cmet.2020.10.026 PMC7858232

[88] E. K. Mitten , G. Baffy , J. Hepatol. 2022, 77, 1642. 36063966 10.1016/j.jhep.2022.08.028

[89] R. K. Moreira , Arch. Pathol. Lab. Med. 2007, 131, 1728. 17979495 10.5858/2007-131-1728-HSCALF

[90] I. Gurevich , S. A. Burton , C. Munn , M. Ohshima , M. E. Goedland , K. Czysz , Biol. Open 2020, 9. 10.1242/bio.055087 PMC7758638 33268331

[91] M. N. B. Ramli , Y. S. Lim , C. T. Koe , D. Demircioglu , W. Tng , K. A. U. Gonzales , C. P. Tan , I. Szczerbinska , H. Liang , E. L. Soe , Z. Lu , C. Ariyachet , K. M. Yu , S. H. Koh , L. P. Yaw , N. H. B. Jumat , J. S. Y. Lim , G. Wright , A. Shabbir , Y. Y. Dan , H.‐H. Ng , Y.‐S. Chan , Gastroenterology 2020, 159, 1471. 32553762 10.1053/j.gastro.2020.06.010

[92] N. F. Boyd , L. J. Martin , M. J. Yaffe , S. Minkin , Breast Cancer Res. 2011, 13, 223. 22114898 10.1186/bcr2942 PMC3326547

[93] T. W. Kragstrup , M. Kjaer , A. L. Mackey , Scand. J. Med. Sci. Sports 2011, 21, 749. 22092924 10.1111/j.1600-0838.2011.01377.x

[94] B. L. LeSavage , D. Zhang , C. Huerta‐López , A. E. Gilchrist , B. A. Krajina , K. Karlsson , A. R. Smith , K. Karagyozova , K. C. Klett , M. S. Huang , C. Long , G. Kaber , C. M. Madl , P. L. Bollyky , C. Curtis , C. J. Kuo , S. C. Heilshorn , Nat. Mater. 2024, 23, 1138. 38965405 10.1038/s41563-024-01908-x PMC13098013

[95] J. G. Roth , M. S. Huang , R. S. Navarro , J. T. Akram , B. L. LeSavage , S. C. Heilshorn , Sci. Adv. 2023, 9, adh8313. 10.1126/sciadv.adh8313 PMC10588948 37862423

[96] B. L. LeSavage , N. A. Suhar , C. M. Madl , S. C. Heilshorn , JoVE 2018, e57739, 10.3791/57739. PMC6101275 29863669

