# Supplementary material for: Engineered Hydrogels for Organoid Models of Human Nonalcoholic Fatty Liver Disease
Source: Adv Sci (Weinh). 2025 May 14;12(22):e17332. doi: 10.1002/advs.202417332 (PMC12165117; doi:10.1002/advs.202417332)
Supplement: Supplementary file 1 — Supporting Information [file ADVS-12-e17332-s001.pdf]

ADVANCED SCIENCE
Open Access

Supporting Information

for *Adv. Sci.*, DOI 10.1002/advs.202417332

Engineered Hydrogels for Organoid Models of Human Nonalcoholic Fatty Liver Disease

*Yueming Liu, Aidan E. Gilchrist, Patrik K. Johansson, Yuan Guan, Jaydon D. Deras, Yu-Chung Liu, Sofia Ceva, Michelle S. Huang, Renato S. Navarro, Annika Enejder, Gary Peltz and Sarah C. Heilshorn**

Supporting Information

Engineered hydrogels for organoid models of human non-alcoholic fatty liver disease

*Yueming Liu[1], Aidan E. Gilchrist[2], Patrik K. Johansson[1], Yuan Guan[3], Jaydon D. Deras[4], Yu-Chung Liu[5], Sofia Ceva[6], Michelle S. Huang[4], Renato S. Navarro[1], Annika Enejder[1], Gary Peltz[3], Sarah C. Heilshorn[1,]***

[1] Department of Materials Science and Engineering, Stanford University, Stanford, CA 94305, USA

[2] Department of Biomedical Engineering, University of California, Davis, Davis 95616, CA, USA

[3] Department of Anesthesiology, Pain and Perioperative Medicine, Stanford University School of Medicine, CA 94305, USA

[4] Department of Chemical Engineering, Stanford University, Stanford, CA 94305, USA

[5] Department of Biomedical Engineering, University of Michigan, Ann Arbor, MI 48109, USA

[6] Department of Biology, Stanford University, Stanford, CA 94305, USA

*Person to whom correspondence should be addressed, heilshorn@stanford.edu

List of Supplemental Information

Figure S1. HA-BZA modification.

Figure S2. ELP-HYD modification.

Figure S3. HOs cultured in HELP with different ELP variants.

Figure S4. HOs cultured in different stiffness HELP matrices on day 16.

Figure S5. HI+ with competitor improved the gel homogeneity and HOs formation.

Figure S6. Representative immunostaining images of HOs cultured in growth media on day 9.

Figure S7. H&E staining of differentiated HOs cultured in different stiffness matrices.

Figure S8. Rhodamine 123 transportation in HOs

Figure S9. HO lipid volume analysis by CARS.

Figure S10. HO mRNA expression in each stiffness matrices with or without OA treatment.

Figure S11. LO and HI+ matrix stiffness with cells on 0 or 16 days of cell culture.

Figure S12. Expression of CYP3A4 in HOs cultured in LO and HI+ HELP matrices.

Figure S13. DCA treatment on HOs cultured in LO and HI+ HELP matrices.

Table S1. List of primer sequences.

Methods S1. Script for CARS analysis.

A

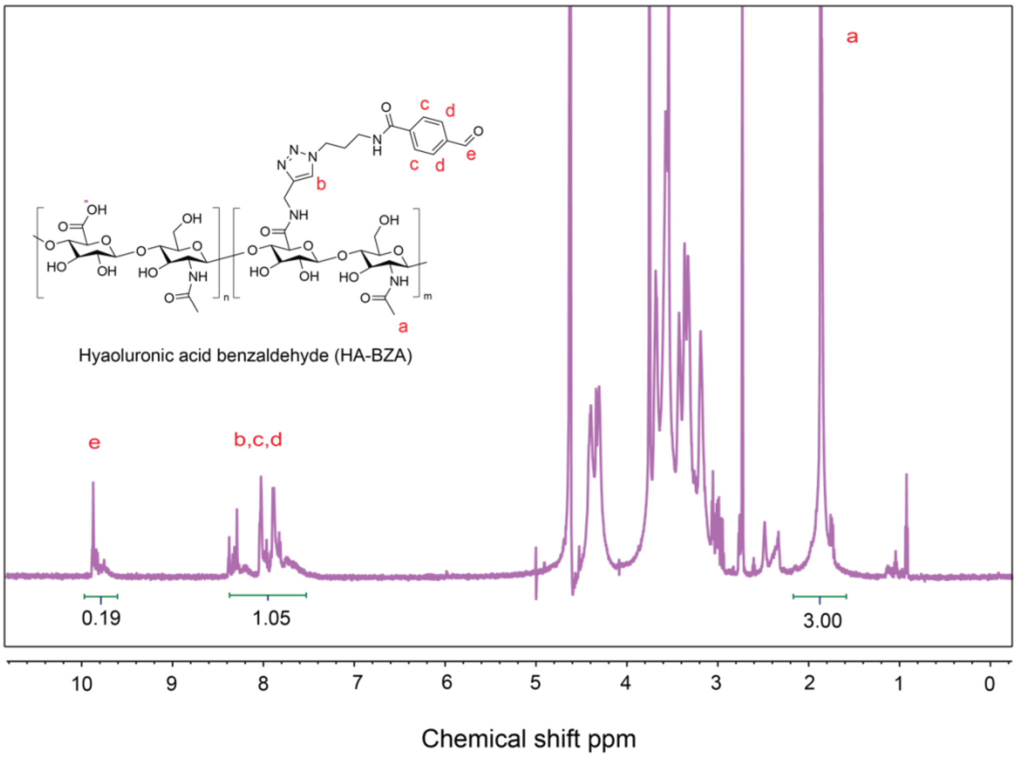

B

$$\text{Modification} = \frac{\text{Acetyl peak H}}{3\text{ H}} \times \frac{\text{aldehyde group H}}{1\text{ H}}$$

$$\text{Modification} = \frac{3\text{ H}}{3\text{ H}} \times \frac{0.19\text{ H}}{1\text{ H}} = 19\%$$

Figure S1. Hyaluronic acid (HA) modification with benzaldehyde functional groups. (**A**) Representative ^{1}H NMR (D_2O solvent) of ~ 19% modified HA-benzaldehyde. (**B**) Calculation of HA- benzaldehyde modification.

A ELP full amino acid sequence

MASMTGGQQMGHHHHHHDDDDKLQLDASTVYAVTG**RGD**SPASSAASAVPGIGVPGIGVPGKGVPGIGVPGIG
VPGIGVPGIGVPGKGVPGIGVPGIGVPGIGVPGIGVPGKGVPGIGVPGIGVPLDASTVYAVTG**RGD**SPASSAASAVP
GIGVPGIGVPGKGVPGIGVPGIGVPGIGVPGIGVPGKGVPGIGVPGIGVPGIGVPGIGVPGKGVPGIGVPGIGVPLD
ASTVYAVTG**RGD**SPASSAASAVPGIGVPGIGVPGKGVPGIGVPGIGVPGIGVPGIGVPGKGVPGIGVPGIGVPGIGV
PGIGVPGKGVPGIGVPGIGVPLDASTVYAVTGRGDSPASSAASAVPGIGVPGIGVPGKGVPGIGVPGIGVPGIGVP
GIGVPGKGVPGIGVPGIGVPGIGVPGIGVPGKGVPGIGVPGIGVPLE*

Tag region; Fibronection-derived integrin binding region; Elastin-like repeat region.

B

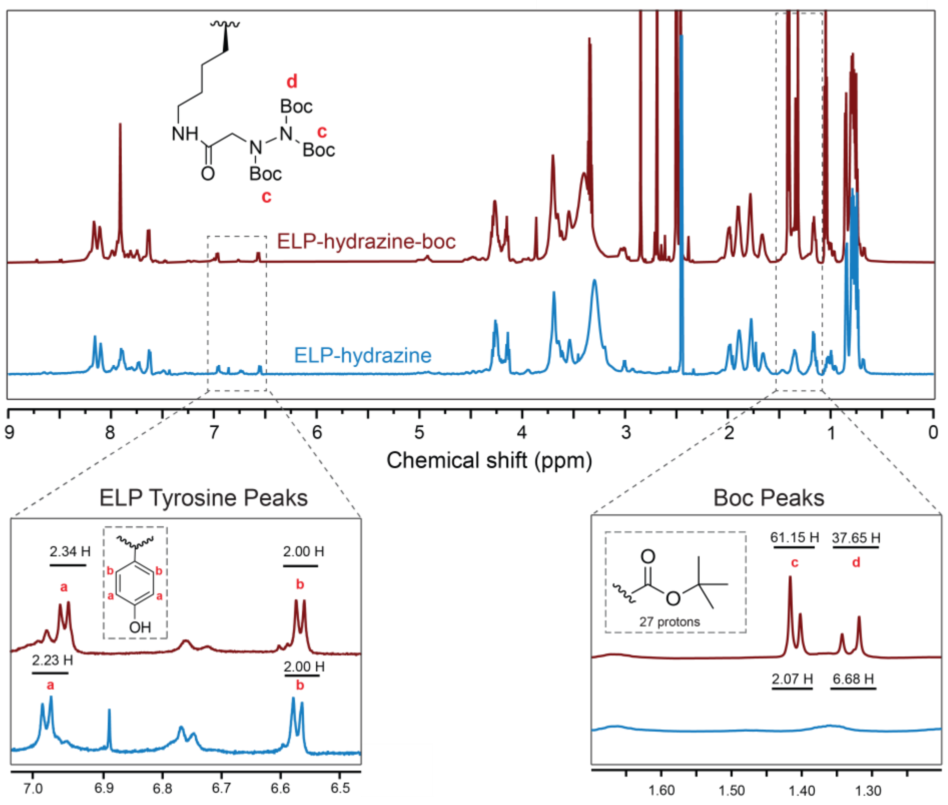

C

$$\frac{\text{Hydrazine groups}}{\text{ELP}} = \frac{\text{Boc peak H}}{\text{Tyrosine peak H}} \times \frac{4\text{ H}}{\text{tyrosine}} \times \frac{4\text{ tyrosine}}{\text{ELP}} \times \frac{1\text{ Boc}}{9\text{ H}} \times \frac{1\text{ hydrazine}}{3\text{ Boc}}$$

$$\frac{\text{Hydrazine groups}}{\text{ELP}} = \frac{98.8\text{ H}}{4.34\text{ H}} \times \frac{4\text{ H}}{\text{tyrosine}} \times \frac{4\text{ tyrosine}}{\text{ELP}} \times \frac{1\text{ Boc}}{9\text{ H}} \times \frac{1\text{ hydrazine}}{3\text{ Boc}} = 13.49$$

$$\frac{13.49}{\text{14 possible hydrazine groups}} = \sim 96\%\text{ modification of ELP}$$

Figure S2. Elastin-like protein (ELP) modification with hydrazine functional groups. (**A**) ELP amino acid sequence. (**B**) Representative ^{1}H NMR (DMSO solvent) of intermediate ELP-hydrazine-Boc (red, top) and final ELP-hydrazine (blue, bottom). The tri-butyloxycarbonyl (tri-Boc) protecting group is completely removed from hydrazine in the final ELP-hydrazine polymer. (**C**) Calculation of ELP hydrazine modification.

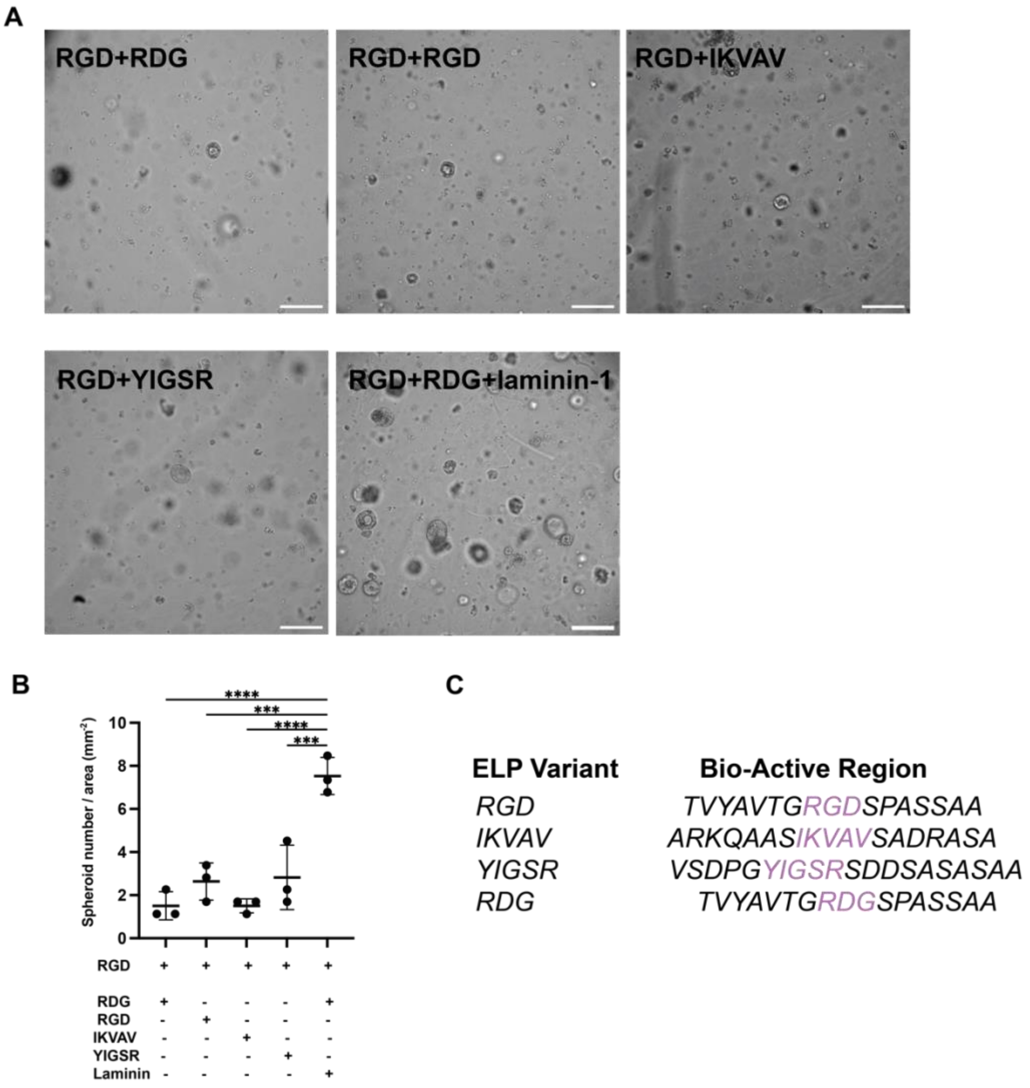

Figure S3. Hepatic spheroids cultured in HELP with different ELP variants. (**A**) Representative bright field images of hepatic spheroids grew in HELP with different ELP variants on Day 7. Scale bar is 100 µm. (**B**) Formation efficiency of spheroids on day 3. Data shown are mean ± sd; Statistical significance was tested by one-way ANOVA with Tukey's multiple comparisons: ***$p<0.001$, ****$p<0.0001$. n = 3 replicate cultures. (**C**) Bio-active sequence in ELP variants.

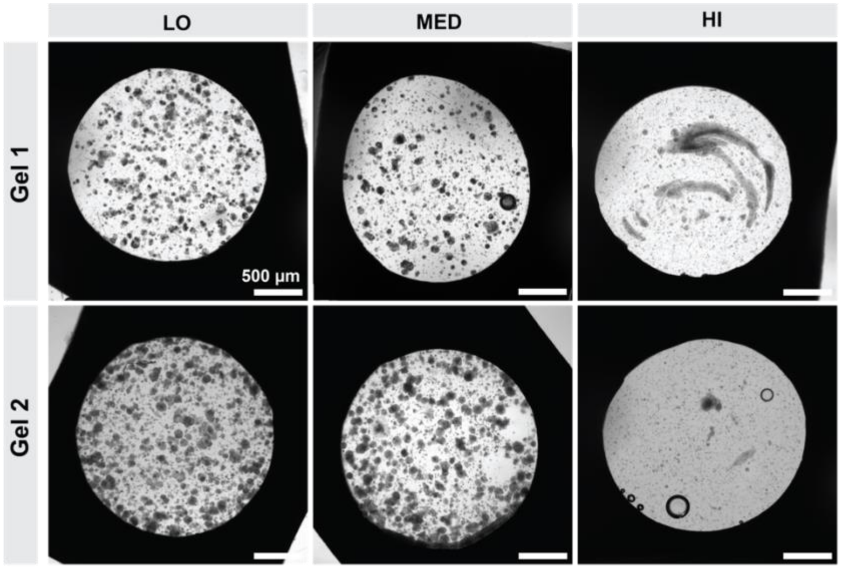

Figure S4. HOs cultured in different stiffness HELP matrices on day 16. HOs showed reproducible growth in LO and MED HELP conditions, while they showed irreproducible growth in HI HELP condition due to high stiffness and rapid gelation.

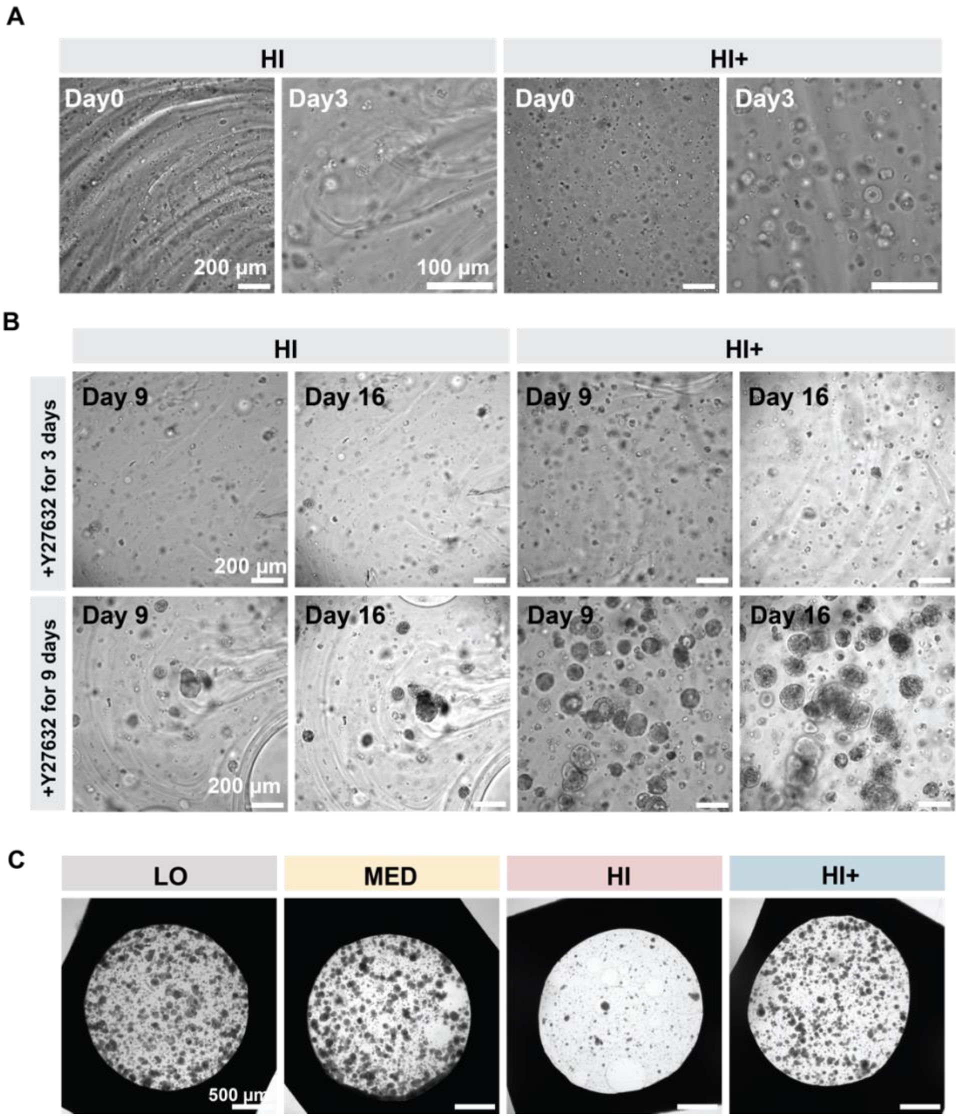

Figure S5. **HI+ with competitor improved the gel homogeneity and HOs formation**. (**A**) Bright field images of HOs grew in HI and HI+ condition on day 1 and day3. HI+ showed more homogeneous mixing and improved organoid formation efficiency on day 3. (**B**) Brightfield images of hepatic spheroids cultured in HI and HI+ with Y27632 added for 3 days or 9 days. Y27632 showed to improve spheroids survival and growth. (**C**) Representative images of HOs cultured in different stiffness HELP matrices. HI+ showed improved HO culture compared to HI, and was comparable to LO and MED.

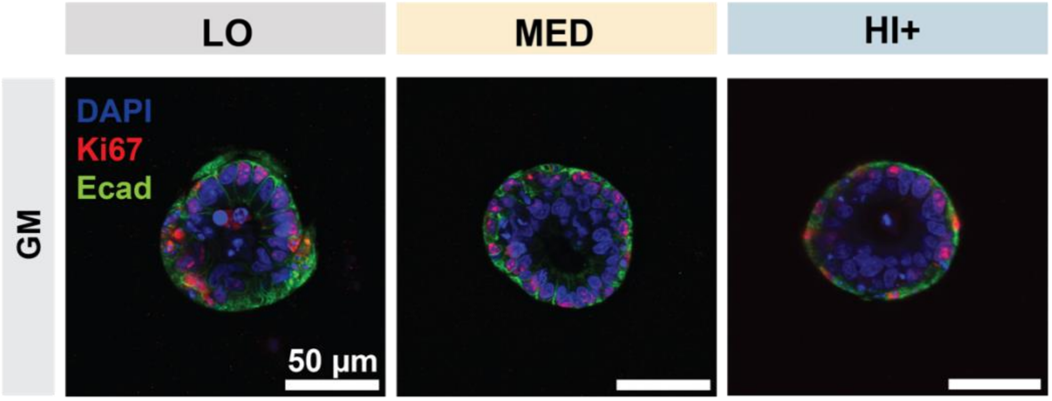

Figure S6. Representative immunostaining images of hepatic spheroids cultured in growth media on day 9. Spheroids showed positive staining for cell proliferation marker (Ki67) and the cell membrane protein E-cadherin.

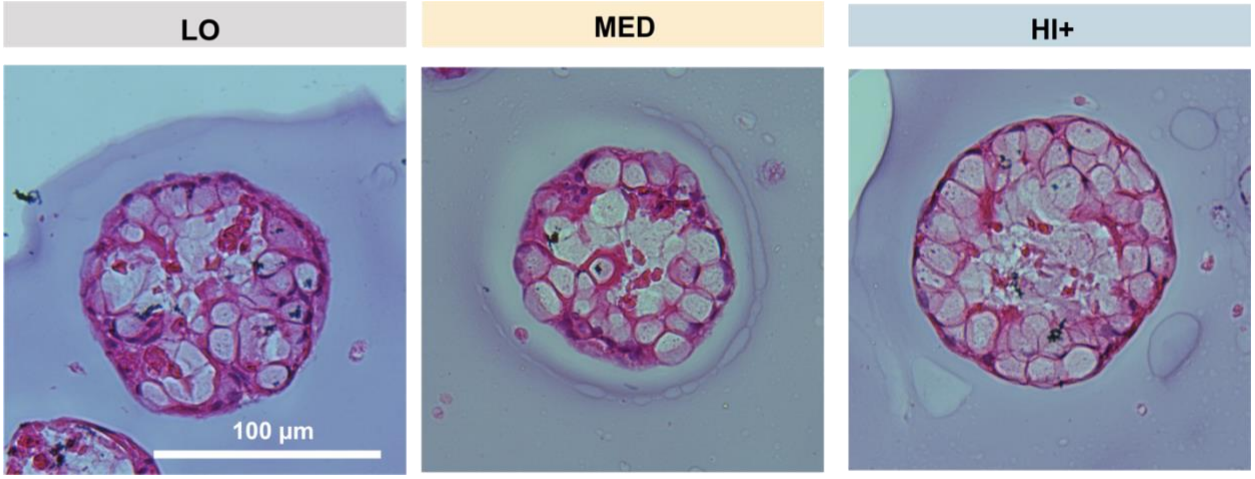

Figure S7. Hematoxylin and Eosin (H&E) staining of differentiated HOs cultured in different stiffness matrices on Day 16. H&E staining showed that HOs cultured in LO, MED, and HI+ stiffness conditions had similar size and mature morphology after the full 16 days.

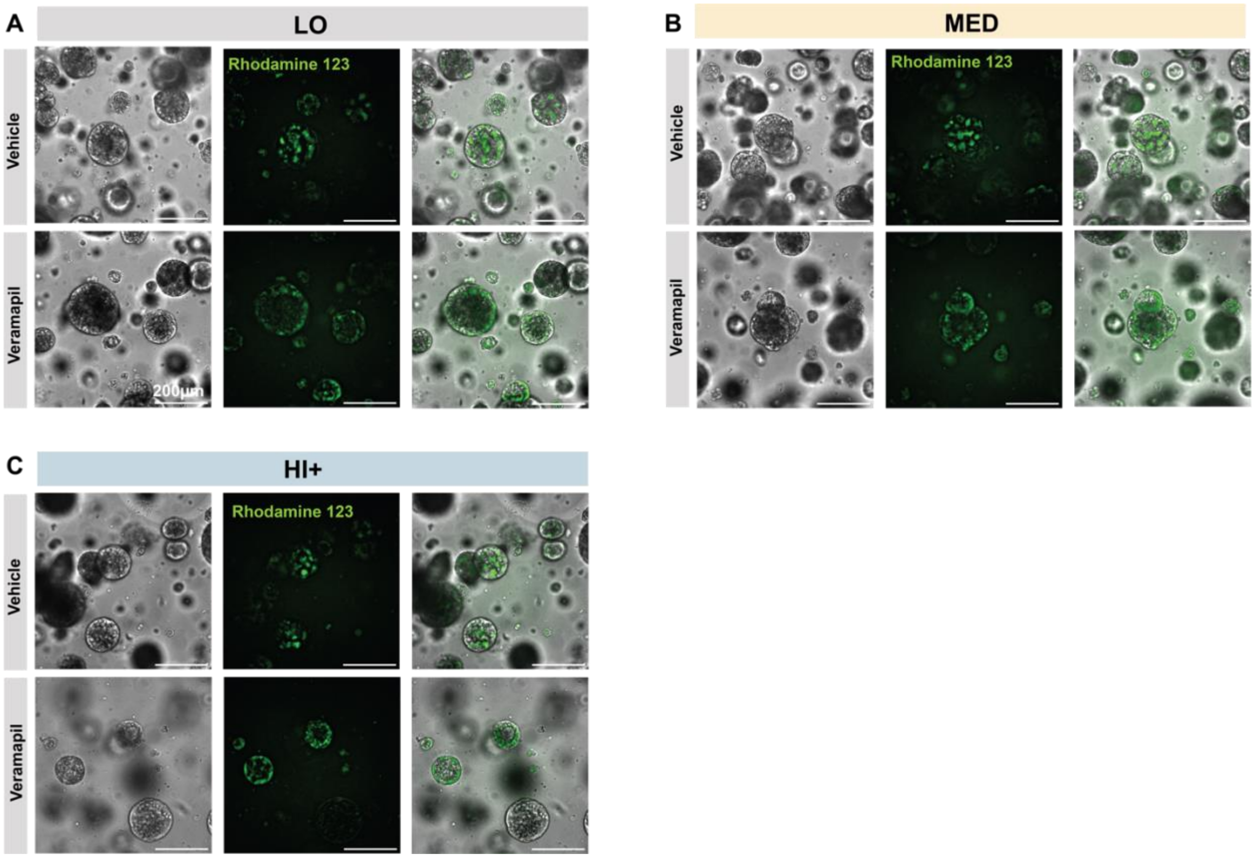

Figure S8. Rhodamine 123 transport in differentiated HOs cultured in different stiffness matrices on Day 16. When pretreated with the multidrug resistant protein 1 (MRP1) inhibitor verapamil, HOs accumulated fluorescence only in the cell cytoplasm, whereas without verapamil treatment (vehicle only), HOs accumulated fluorescence in the organoid lumens, indicating successful MRP1 transport function.

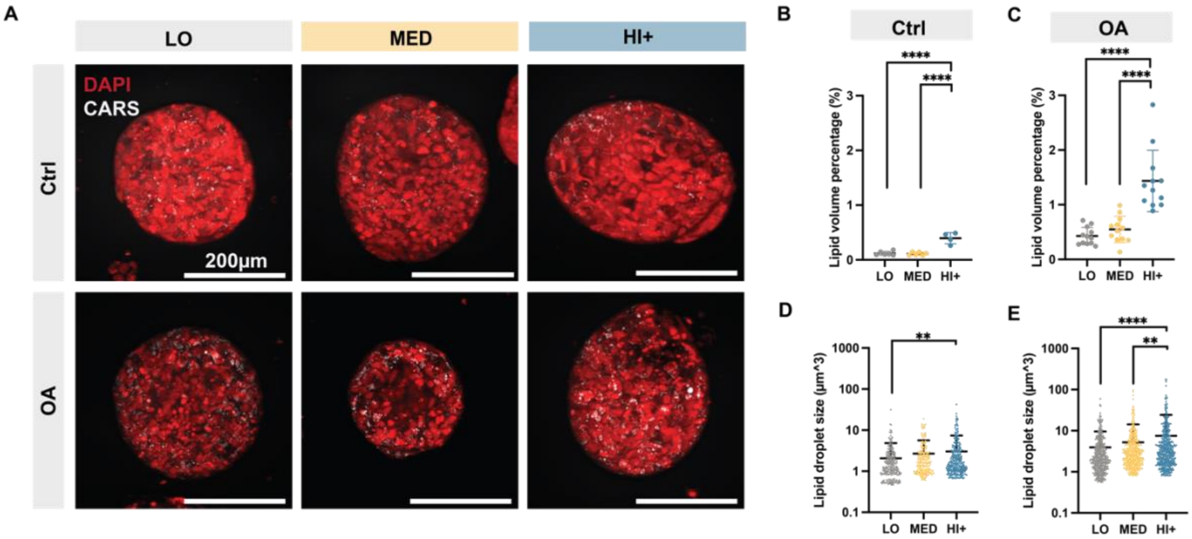

Figure S9. HOs lipid volume analysis by CARS. (**A**) Colocalization fluorescence and CARS images show lipid droplets in HOs cultured in different stiffness matrices. (**B-C**) Quantification of lipid volume percentage of organoids without OA treatment (B) or with OA treatment (C). (**D-E**) Quantification of individual lipid droplet size of organoids without OA treatment (D) or with OA treatment (E). Data shown are mean ± sd; Statistical significance was tested by one-way ANOVA with Tukey's multiple comparisons: $^{**}p<0.01$, $^{***}p<0.001$, $^{****}p<0.0001$. n = 4 replicate cultures with N = 4-11 organoids.

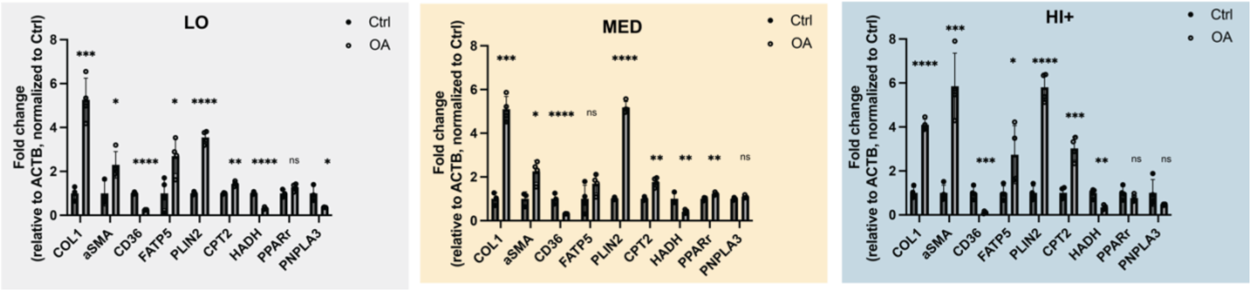

Figure S10. HO mRNA expression in different stiffness matrices with or without OA treatment. Data shown are mean ± sd; Statistical significance was tested by unpaired student t-test: *p<0.05, **p<0.01, ***p<0.001, ****p<0.0001, n = 3-4 replicate cultures.

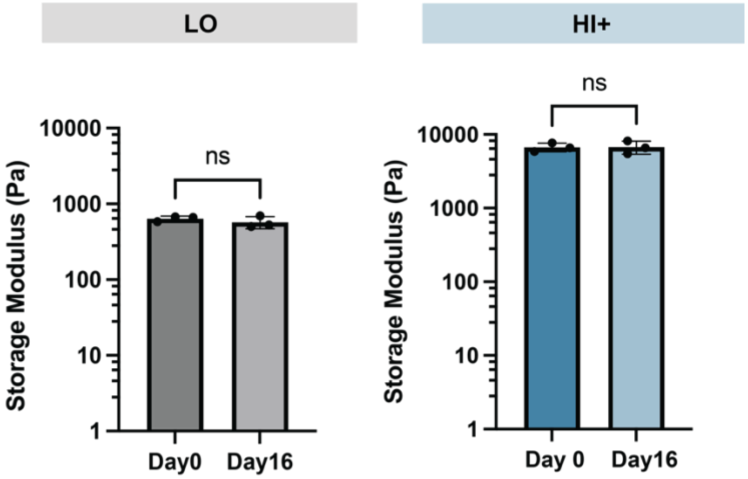

Figure S11. LO and HI+ matrix stiffness after 0 or 16 days of HO culture. HOs did not change matrix stiffness over 16 days of cell culture**.** Data shown are mean ± sd; statistical significance was tested by unpaired student t-test; ns: not significant; n = 3 replicate cultures.

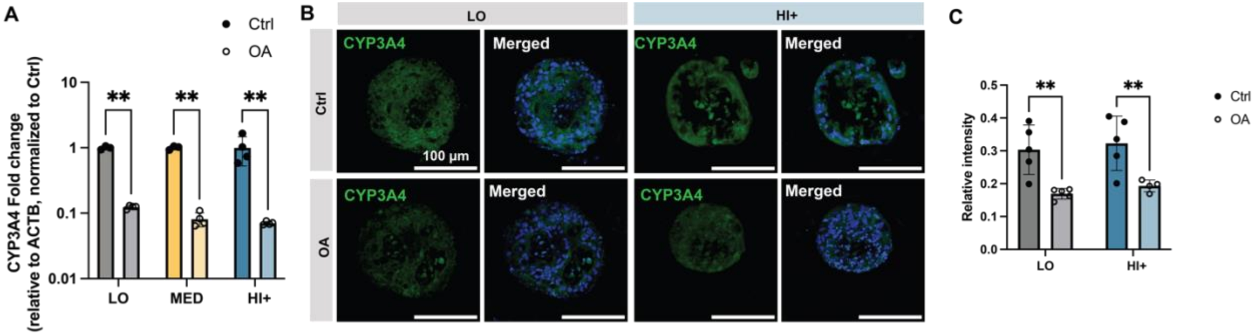

Figure S12. Expression of CYP3A4 in HOs cultured in LO and HI+ HELP matrices. (**A**) mRNA expression of CYP3A4 in different stiffness matrices with or without OA treatment. Data shown are mean ± sd; Statistical significance was tested by unpaired student t-test: $^{**}p<0.01$, n = 3-4 replicate cultures. (**B**) Representative immunofluorescence images of CYP3A4 in HOs cultured in HELP LO and HELP HI+ with 0 μM or 500 μM OA treatment. (**C**) Quantification of CYP3A4 in HOs cultured in HELP LO and HELP HI+. Data shown are mean ± sd; Statistical significance was tested by unpaired student t-test: $^{**}p<0.01$, n = 3-4 replicate cultures with N = 4-5 organoids.

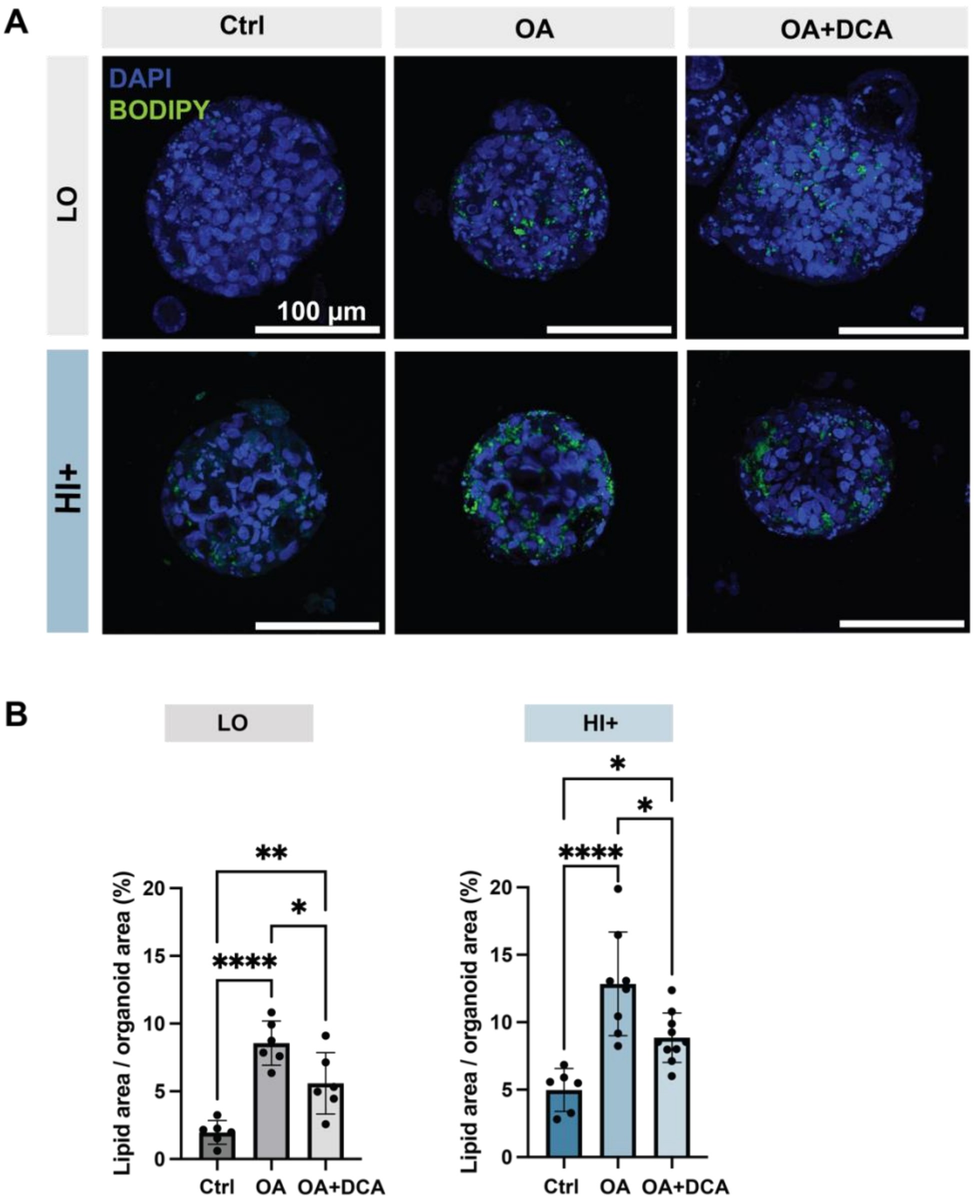

Figure S13. DCA treatment on HOs cultured in LO and HI+ HELP matrices. (**A**) Representative lipid accumulation images of HOs cultured in LO and HI+ stiffness matrices with no treatment (Ctrl), OA treatment or OA+ 20 μM DCA treatment. (**B**) Quantification of lipid area as percentage of organoid area in HELP LO (left) and HI+ (right) matrices. Data shown are mean ± sd; Statistical significance was tested by one-way ANOVA with Tukey's multiple comparisons: *p<0.05, **p<0.01, ***p<0.001, ****p<0.0001. n = 3-4 replicate cultures with N = 6-8 organoids.

Table S1. List of primer sequences.

Target	Forward primer (5' to 3')	Reverse primer (5' to 3')
β-actin	CACCATTGGCAATGAGCGGTTC	AGGTCTTTGCGGATGTCCACGT
COL-1	GAGAGCATGACCGATGGATT	ATGTAGGCCACGCTGTTCTT
α-SMA	CCGACCGAATGCAGAAGGA	ACAGAGTATTTGCGCTCCG
CD36	CTTTGGCTTAATGAGACTGGGAC	GCAACAAACATCACCACACCA
FATP5	ACACACTCGGTGTCCCTTTC	CTACAGGGCCCACTGTCATT
HADH	TGTCGGACTGGATACTACGA	GATGGGCTGGGCTGATGTAA
CPT2	TGATGGTTGAGTGCTCCAAGT	AAGTGTCGGTCAAAGCCCTG
PLIN2	GATGGCAGAGAACGGTGTGAAG	CAGGCATAGGTATTGGCAACTGC
PNPLA3	AGTCGTGGATGCCTTGGTATG	CGGTGATGGTTGTTTTGGCA
PPARγ	AGCCTGCGAAAGCCTTTTGGTG	GGCTTCACATTCAGCAAACCTGG

Methods S1. Scripts for CARS lipid analysis

```
// 1. Update input parameters, result folder etc below

// 2. Place all spectral files and the power file in the "folder" defined below (specify)

// 3. Open the fluorescence stack with the nuclei, cell marker and cell expression images.

// 4. Check that its number of slides matches that of the spectral files. Else the last slides are truncated.

// Output: a table with spectra for each lipid aggregate

// Output: arrays with particle volumes, whether intra/extracellular and collocalizing with the cell expression marker

// Output: multiphannel image with the cell outlines, thresholded CARS lipid aggregates and regions identified by the cell expression marker

macro "SpectraLipidsInLabeledRegions" {

				//*****Input Data

				// The input data in a folder with spectral files, typically 1-32

				close("*");

		folder = getDirectory("Choose Data Directory");

		// Output folder where result data and images are stored

		resultFolder = getDirectory("Choose Results Directory");

	fileID="HO-HI_ctrl";
```

```
	result_file=resultFolder + "Spectra_" + fileID + ".csv";


		//Specifies which multipoint series in the spectral files to be evaluated; 0 means no multipoint acquisition
		multipoint=0;
		//Substring in the fluorescence file name to be able to find it
		nucleiChannel=1; //Channel in the stack that represent the nuclei
		proteinChannel=2; //Channel in the stack that represent a cell expression, e.g. Actin
		CARSChannel=3; //Channel in the CARS stack that represent the lipids


		//Generated a lipid mask from the stack mask_stack, typically the  2845 cm-1 image, Image 9
		//Form lipid ratio image between vibration measured in file 9 (2845 cm-1) and file 14/17/19 (2880/2911/2930 cm-1)
		mask_stack=9; //7970
		numerator=9; //7915
		denominator=19;


		//Output table
		Table.create("Spectra");


		calibrationlist=power_list(folder);
		//List of the spectral files
	CARSimagelist=generate_Nikonfilelist(folder);


//	open(directoryID + "Reference.tif");
//		selectWindow("Reference.tif");
```

```
//			REF_image=getTitle();



			//*****End Input Data



			//*****Fluorescence stack masks: nuclei, cell & cell expression masks
			//Assume that the fluorescence stack is open
		//openimagelist=getList("image.titles");
		//Find the fluorescence stack
			//i=0;
			//while ((indexOf(openimagelist[i], cellMarker) < 0) && (i <
openimagelist.length)) {
				//Close all other images in order not to conflict with the spectral analysis
				//close(openimagelist[i]);
				//i=i+1;
			//}
			//if (i==openimagelist.length) {
				//exit("No fluorescence image open");
			//}
			//Creates the nucleiMask, cellMask and proteinMask
			//fluorescence_masks(openimagelist[i], nucleiChannel, cellMarkerChannel,
proteinChannel);

			//*****End Fluorescence stack masks: nuclei, cell & cell expression masks
```

```
//*****Lipid mask from the mask stack
RawStack=CARSimagelist[(mask_stack-1)];
file=folder + RawStack;
print(file);

if (multipoint==0) {
        run("Bio-Formats Importer", "open=file autoscale color_mode=Default
specify_range view=Hyperstack stack_order=XYCZT c_begin=3 c_end=3 c_step=1");
}
else {

}


rename(RawStack);
selectWindow(RawStack);

//The CARS raw stack as initial data
run("Duplicate...", "duplicate channels=" + CARSChannel);
rename("CARSStack");

//run("East", "stack");
//imageCalculator("Divide stack", "CARSStack", REF_image);
run("Gaussian Blur...", "sigma=1.2 stack");
run("Subtract Background...", "rolling=30 stack");
```

```
		//**Generates a 3D mask ("3DLipidMask") of the lipid/protein particles in the entire stack
		//And a labeled stack LabeledObjects, as well as its max projection ObjectProjection
		//Prints an array with the volumes of each lipid object in the table Spectra
		generate_lipidmasks("CARSStack");

		//*****End Lipid mask from the mask stack




		//Colocalize Lipid objects with the cell and protein masks
		//Print arrays in the table Spectra; yes:>0 no:0 for each labeled lipid object
		//coloc_cells_proteins();




		//*****Evaluate all spectral files
		//No Background subtraction
		close("Results");
		//For each vibration
		for (series=0; series< CARSimagelist.length; series++) {

			file=folder + CARSimagelist[series];

			//Open the CARS stack only
			if (multipoint==0) {
```

```
			run("Bio-Formats Importer", "open=file autoscale color_mode=Default specify_range view=Hyperstack stack_order=XYCZT c_begin=3 c_end=3 c_step=1");
		}
		else {

		}
		rename("Lipids");
		run("Divide...", "value="+calibrationlist[series]+" stack");
		//run("East", "stack");
		//imageCalculator("Divide stack", "Lipids", REF_image);
		run("Gaussian Blur...", "sigma=0.8 stack");
		//run("Subtract Background...", "rolling=30 stack");
		//run("Add...", "value=0 stack");

		//Evaluate the average intensity for each 3D object in each cell defined by the "3DLipidMask"
		//Results printed in the table Spectra
		evaluate_Stack(series);

		if (series==numerator-1){
			imageCalculator("Multiply create 32-bit stack", "Lipids","3DLipidMask");
			run("Divide...", "value=255 stack");
			rename("numerator_stack");
		}
		if (series==denominator-1){
			imageCalculator("Multiply create 32-bit stack", "Lipids","3DLipidMask");
```

```
                run("Divide...", "value=255 stack");
                rename("denominator_stack");

            }
            close("Lipids");

        }
        //*****End Evaluate all spectral files



        //******Output
        //Generate an overview of the numbered objects as 2D max projection
        numbered_overview();

        selectWindow("Spectra");
        Table.save(result_file);




    //Generate the lipid ratio image
        close("Results");
        lipidratio_image();

        //selectImage("nucleiMask");
        //run("Duplicate...", "duplicate");
        //run("32-bit");
        //selectImage("CellOutlines");
```

```
		//run("32-bit");


		//run("Merge Channels...", "c1=nucleiMask-1 c2=CellOutlines c3=MeanLR_image create");
		//rename("CellOutline_LRStack");
		//saveAs("tiff", resultFolder + "CellOutline_LRStack"+fileID);
		//close("CellOutlines");


		//run("Merge Channels...", "c1=nucleiMask c2=cellMask c3=proteinMask c4=3DLipidMask create");
		//rename("Masks");
		//saveAs("tiff", resultFolder + "Masks"+fileID);



		selectImage("LabeledObjects");
		saveAs("tiff", resultFolder + "LabeledObjects3D_"+fileID);
		selectImage("ObjectProjection");
		saveAs("tiff", resultFolder + "NumberedLipids2D_"+fileID);
		selectImage("LR_image");
		saveAs("tiff", resultFolder + "LR_Stack_"+fileID);
		selectImage("MeanLR_image");
		saveAs("tiff", resultFolder + "MeanLR_image_"+fileID);
		selectImage("3DLipidMask");
		saveAs("tiff", resultFolder + "3DLipidMask_"+fileID);
		close("*");
		close("Spectra");
}
```

```
//runMacro(path+"macro collection.ijm", "first_macro");






//***********************************************
function power_list(path) {

	filelist = getFileList(path);
	powerList=newArray(filelist.length);
	for (i = 0; i < filelist.length; i++) {
		if (indexOf(filelist[i], ".txt") >= 0) {
			break;
		}
	}
	if (i==(filelist.length)) {
		print("No power calibration file in " + path);
		for (vibration = 0; vibration < filelist.length; vibration++) {
			powerList[vibration]=1;
		}
	}
	else {
		file=path+filelist[i];
		Table.open(file);
		powerList=Table.getColumn("Power");
		run("Close");
```

```
        Array.getStatistics(powerList, min, max, mean, stdDev);
        for (i = 0; i < powerList.length; i++) {
            powerList[i]=(powerList[i]*powerList[i])/(mean*mean);
        }
    }
    return powerList;
}


//********************************************
function generate_Nikonfilelist(path) {

    filelist = getFileList(path);
    ilist=newArray(filelist.length);



    //Nikon files only
    index=0;
    for (i = 0; i < filelist.length; i++) {
        if (indexOf(filelist[i], ".nd") >= 0) {
            ilist[index]=filelist[i];
            index=index+1;
        }
    }
    //ilist was originally an array with zeros of the same length as filelist
    //elements not assigned are deleted
    ilist=Array.deleteValue(ilist, 0);
```

```
	//Make sure that the files are listed in the order they are collected
	//Assume that they are numbered
	ilist=Array.sort(ilist);
	return ilist;
}


//*********************************************
function fluorescence_masks(fluorescenceStack, Nuc_channel, Prot_Channel) {
		selectWindow(fluorescenceStack);
		run("Duplicate...", "duplicate channels=" + Nuc_channel);
		rename("nucleiMask");
		run("Gaussian Blur...", "sigma=5 stack");
		setAutoThreshold("Triangle dark no-reset stack");
		waitForUser("Optimize threshold and press OK");
		run("Convert to Mask", "method=Triangle background=Dark black");
		run("Fill Holes", "stack");
		//Remove sub-nuclear sized particles <5 micron2
		run("Analyze Particles...", "size=5-Infinity show=Masks include stack");
		run("Invert LUT");
		run("Blue");

		selectWindow(fluorescenceStack);
		run("Duplicate...", "duplicate channels=" + Cell_Channel);
		rename("cellMask");
```

```
	midslice=round(nSlices/2);
setSlice(midslice);
	run("Gaussian Blur 3D...", "x=3 y=3 z=2");
	setAutoThreshold("Triangle dark no-reset stack");
	waitForUser("Optimize threshold and press OK");
	run("Convert to Mask", "method=Triangle background=Dark black");
	//Remove noise <1 micron2 in area
	run("Analyze Particles...", "size=1-Infinity show=Masks include stack");
	run("Invert LUT");
	run("Cyan");

	//Cell outlines
	run("Duplicate...", "duplicate");
	run("Grays");
	run("Find Edges", "stack");
	rename("CellOutlines");

	selectWindow(fluorescenceStack);
	run("Duplicate...", "duplicate channels=" + Prot_Channel);
	rename("proteinMask");
	midslice=round(nSlices/2);
setSlice(midslice);
	run("Gaussian Blur...", "sigma=3 stack");
	setAutoThreshold("Intermodes dark no-reset stack");
	waitForUser("Optimize threshold and press OK");
	run("Convert to Mask", "method=Intermodes background=Dark black");
	run("Red");
```

```
}

//******************************************

function generate_lipidmasks(carsStack) {
	selectWindow(carsStack);
	//Refers to "CARSStack"
	midslice=round(nSlices/2);
   setSlice(midslice);
   resetMinAndMax();
   setAutoThreshold("Intermodes dark no-reset stack");
   //setAutoThreshold("Intermodes dark stack");
	waitForUser("Optimize threshold and press OK");
	run("Convert to Mask", "background=Dark black");
	waitForUser("Check Mask");
	//Separate 3D particles with disconnection=0 to 1 - 0 means no disconnection and 1 high degree of disconnection
	run("Disconnect Particles", "disconnection=0.4000 xsize=1.0000 ysize=1.0000 zsize=1.0000 algorithm=[new algorithm] euler=26 sigma=1,1,1 distance=0.5000 holes,=0.0000 particles,=0.0000 separate entire evaluation=[3D volumetric processing]");
	//Results in a labeled stack
	rename("DisconnectedLipidMask");
	//Stack.getStatistics(voxelCount, mean, min, max, stdDev);
	//Filters out the smallest objects and generates a labeled stack
	//Generates a table "Statistics for DisconnectedLipidMask" with a column with particle volumes
```

```
run("3D OC Options", "volume dots_size=5 font_size=30 store_results_within_a_table_named_after_the_image_(macro_friendly) redirect_to=none");
run("3D Objects Counter", "threshold=1 slice=9 min.=150 max.=19922944 objects statistics");
rename("LabeledObjects");
run("Enhance Contrast", "saturated=0.35");
run("Fire");

run("Duplicate...", "duplicate" );
rename("3DLipidMask");
close("DisconnectedLipidMask");

//Turn the labeled stack into a mask
selectImage("3DLipidMask");
//setAutoThreshold("MinError dark");
setThreshold(1, 65535);
setOption("BlackBackground", true);
run("Convert to Mask", "method=MinError background=Dark black");
run("Yellow");
//No need for the CARS stack of the mask file
close(carsStack);

selectImage("LabeledObjects");
run("Z Project...", "projection=[Max Intensity]");
rename("ObjectProjection");
run("32-bit");

selectWindow("Statistics for DisconnectedLipidMask");
```

```
	Volume=Table.getColumn("Volume (micron^3)");
	close("Statistics for DisconnectedLipidMask");
	selectWindow("Spectra");
	Table.setColumn("Volume", Volume);

}

//********************************************
function evaluate_Stack(series) {
	//Evaluation of all lipid objects for one vibration
	//Initialize local arrays for all particle intensity means (one vibration)
	Mean=newArray(0);


	//In MorphoLibJ plugin library
	run("Intensity Measurements 2D/3D", "input=Lipids labels=LabeledObjects mean");
	selectWindow("Lipids-intensity-measurements");
	Mean=Table.getColumn("Mean");
	close("Lipids-intensity-measurements");


	selectWindow("Spectra");
	Table.setColumn("Vibr." + series, Mean);

}

//********************************************
```

```
function coloc_cells_proteins(){
		//Make sure that the fluorescence stack masks cover the same depth as the CARS
stacks
		selectWindow("3DLipidMask");
		CARSSlices=nSlices;
		selectWindow("cellMask");
		FluorSlices=nSlices;
		//If not the same number of slices
		if (FluorSlices!=CARSSlices) {
			//start=((FluorSlices-CARSSlices)/2+1);
			//end=(FluorSlices-CARSSlices)/2+CARSSlices;
			start=1;
			end=CARSSlices;
			selectWindow("nucleiMask");
			run("Duplicate...", "duplicate range=" + start +"-"+ end);
			close("nucleiMask");
			selectWindow("nucleiMask-1");
			rename("nucleiMask");

			selectWindow("cellMask");
			run("Duplicate...", "duplicate range=" + start +"-"+ end);
			close("cellMask");
			selectWindow("cellMask-1");
			rename("cellMask");

			selectWindow("CellOutlines");
			run("Duplicate...", "duplicate range=" + start +"-"+ end);
```

```
		close("CellOutlines");
		selectWindow("CellOutlines-1");
		rename("CellOutlines");

		selectWindow("proteinMask");
		run("Duplicate...", "duplicate range=" + start +"-"+ end);
		close("proteinMask");
		selectWindow("proteinMask-1");
		rename("proteinMask");
	}

	selectWindow("LabeledObjects");
	Stack.getStatistics(voxelCount, mean, min, LabelMax, stdDev);
	histoMax=LabelMax+1;

	k1=1/255;
	run("Calculator Plus", "i1=cellMask i2=LabeledObjects operation=[Multiply: i2 =
(i1*i2) x k1 + k2] k1="+ k1 +" k2=0 create");
	//imageCalculator("AND create stack", "LabeledObjects","cellMask");
	rename("Labeled_LipidsInCells");
	run("16-bit");
	CellLipids=newArray(histoMax);
	for (i = 1; i <= nSlices; i++) {
	setSlice(i);
	getHistogram(values, slideHistogram, histoMax, 0, histoMax);
	for (bin = 0; bin < histoMax; bin++) {
			CellLipids[bin]+=slideHistogram[bin];
```

```
			}
		}

		close("Labeled_LipidsInCells");

		run("Calculator Plus", "i1=proteinMask i2=LabeledObjects operation=[Multiply: i2 = (i1*i2) x k1 + k2] k1="+ k1 +" k2=0 create");
		//imageCalculator("AND create stack", "LabeledObjects","proteinMask");
		rename("Labeled_LipidsColocProteins");
		ProteinLipids=newArray(histoMax);
		for (i = 1; i <= nSlices; i++) {
		setSlice(i);
		getHistogram(values, slideHistogram, histoMax, 0, histoMax);
		for (bin = 0; bin < histoMax; bin++) {
				ProteinLipids[bin]+=slideHistogram[bin];
			}
		}

		close("Labeled_LipidsColocProteins");

		//First elements represent the background, surrounding the lipid objects
		CellLipids = Array.slice(CellLipids,1);
		ProteinLipids = Array.slice(ProteinLipids,1);

		selectWindow("Spectra");
		Table.setColumn("In_cell", CellLipids);
		Table.setColumn("Protein_Coloc", ProteinLipids);
```

```
}

//******************************************

function numbered_overview(){
	//An overview map of the numbered objects

	selectImage("ObjectProjection");

	getPixelSize(unit, pixelWidth, pixelHeight);
	//In MorphoLibJ plugin library
	run("Analyze Regions", "centroid");
	//The output table with Centroid X and Centroid Y values for each object
	selectWindow("ObjectProjection-Morphometry");
	ObjectNr=Table.getColumn("Label");
	ObjectX=Table.getColumn("Centroid.X");
	ObjectY=Table.getColumn("Centroid.Y");
	num_objects=Table.size;
	setFont("SansSerif", 20);
	selectImage("ObjectProjection");
	for (object = 1; object <= num_objects; object++) {
		//drawString(object, (ObjectX[object-1]/pixelWidth), (ObjectY[object-
1]/pixelHeight));
		drawString(ObjectNr[object-1], (ObjectX[object-1]/pixelWidth),
(ObjectY[object-1]/pixelHeight));
```

```
	}
	close("ObjectProjection-Morphometry");

}


//**********************************************
function lipidratio_image() {

	imageCalculator("Divide create 32-bit stack", "numerator_stack","denominator_stack");
	rename("LR_image");

	//Create average map of numerator
	selectImage("numerator_stack");
	run("Duplicate...", "duplicate");
	run("Intensity Measurements 2D/3D", "input=numerator_stack-1 labels=LabeledObjects
mean");
	selectImage("LabeledObjects");
	call("inra.ijpb.plugins.LabelToValuePlugin.process", "Table=numerator_stack-1-
intensity-measurements", "Column=Mean", "Min=0", "Max=2500");
	rename("LR_numerator");
	close("numerator_stack-1");
	close("numerator_stack-1-intensity-measurements");

	//Create average map of denominator
	selectImage("denominator_stack");
	run("Duplicate...", "duplicate" );
```

```
	run("Intensity Measurements 2D/3D", "input=denominator_stack-1 labels=LabeledObjects mean");
	selectImage("LabeledObjects");
	call("inra.ijpb.plugins.LabelToValuePlugin.process", "Table=denominator_stack-1-intensity-measurements", "Column=Mean", "Min=0", "Max=2500");
	rename("LR_denominator");
	close("denominator_stack-1");
	close("denominator_stack-1-intensity-measurements");

	//Create ratio map of average numerator and denominator maps
	imageCalculator("Divide create 32-bit stack", "LR_numerator", "LR_denominator");
	rename("MeanLR_image");
	close("LR_numerator");
	close("LR_denominator");
	setMinAndMax(0.4, 0.8);
	run("Red Hot");




	selectWindow("LR_image");
	setMinAndMax(0.4, 0.8);
	run("Red Hot");


	close("numerator_stack");
	close("denominator_stack");
}
```

```
//close("*");
//for (slice = 1; slice<=nSlices; slice++){
        //setSlice(slice);
        //changeValues(250,255,0);
//}
```
